# Utilizing native nanodiscs to isolate active TRPC3 channels and expand structural analysis capabilities

**DOI:** 10.1038/s41598-025-13218-6

**Published:** 2025-08-05

**Authors:** Jasmin Baron, Lena Bauernhofer, Sandro Keller, Franz P. W. Radner, Carolyn Vargas, Gerd Leitinger, Lukas Bernauer, Anita Emmerstorfer-Augustin, Patrick Wiedner, Grégory Durand, Marine Soulié, Victoria Dorrer, Matthias Schittmayer, Ruth Birner-Gruenberger, Michaela Lichtenegger, Matthias Gsell, Klaus Groschner, Rainer Schindl, Oleksandra Tiapko

**Affiliations:** 1 Gottfried Schatz Research Center, Division of Medical Physics and Biophysics, Medical University of Graz, Graz, Austria; 2 Biophysics, Institute of Molecular Biosciences (IMB), NAWI Graz, University of Graz, Graz, Austria; 3 Field of Excellence BioHealth, University of Graz, Graz, Austria; 4 BioTechMed-Graz, Graz, Austria; 5 Institute of Molecular Biosciences, Division of Biochemistry, University of Graz, Graz, Austria; 6 Gottfried Schatz Research Center, Division of Cell Biology, Histology and Embryology, Medical University of Graz, Graz, Austria; 7 Institute of Molecular Biotechnology, Graz University of Technology, Graz, Austria; 8 Avignon Université, UPRI, Avignon Cedex 9, 84916 France; 9 Avignon Université, Chem2staB, Avignon Cedex 9, 84916 France; 10 Institute of Chemical Technologies and Analytics, Technische Universität Wien, Getreidemarkt 9, Vienna, 1060 Austria; 11 Diagnostic and Research Institute of Pathology, Medical University of Graz, Stiftingtalstraße 6, Graz, 8036 Austria; 12 12 MEFOgraz, Graz, Austria

**Keywords:** TRPC3, Protein purification, Nanodiscs, Reconstitution, Electrophysiology, Transient receptor potential channels, Single-channel recording, Biochemical assays

## Abstract

Recent advances in structural biology have provided insights into TRPC3, a TRP family member involved in various (patho)physiological processes. However, the lack of structural information on the channel’s open pore hampers understanding of its function and therapeutic potential. Cryogenic electron microscopy holds promise for elucidating TRPC3’s open-pore conformation, but challenges remain in isolating it without compromising function. Our study evaluated novel extraction agents in comparison to conventional detergents for isolating functional TRPC3 complexes from HEK293, *Komagataella phaffii*, and Expi293F cells, identifying Expi293F as optimal for TRPC3 expression. Among the extraction agents screened, dodecyl diglucoside (DDDG) and *n*-dodecyl-β-D-maltoside (DDM) were the most effective for extracting TRPC3. We successfully purified TRPC3 under native conditions, preserving its tetrameric structure and activity, as confirmed by electron microscopy, mass spectrometry and patch-clamp analysis. This study highlights the importance of extraction agents in advancing TRPC3 research and therapeutic development.

**Supplementary Information:**

The online version contains supplementary material available at 10.1038/s41598-025-13218-6.

## Introduction

The canonical transient receptor potential channel 3 (TRPC3) is a member of the TRP family that displays substantial constitutive activity in both excitable and non-excitable cells. Functionally, TRPC3 is involved in transducing signals downstream of G protein-coupled receptors^1–7^. The main activation mechanism of TRPC3 arises from a direct interaction with diacylglycerol (DAG), which involves a lateral gating fenestration of the pore domain^8–19^. The widespread expression of TRPC3 channels in both excitable and non-excitable cells underscores their central involvement in various physiological and pathological processes^18,20–27^, including tumors, cardiac arrhythmias^28–31^, and neuropathologic processes^32–41^.

Recent advances in single-particle cryogenic electron microscopy (cryo-EM) have provided high-resolution structural models of TRPC3 complexes, shedding light on their regulation^15,16,42^. However, existing models lack critical details of the open-pore structure, hampering a comprehensive understanding of TRPC3 physiology and impeding the development of therapeutic ligands. Analysis of lipid dependency of TRPC3 activity has revealed associations with diacylglycerol, cholesterol, and PIP_2_^10,12–18,43–45^, indicating that protein lipidation is crucial for TRPC3 activity and emphasizing the significance of the lipid enviroment in structural studies. Thus, protein extraction from the native lipid environment can compromise ion-channel conformation, resulting in a closed pore even in the presence of agonists. On a broader scale, understanding the structures, dynamics, and functions of membrane proteins is challenging due to their hydrophobic nature and the complex composition of cellular membranes, which present numerous interaction partners^46–49^. Extracting membrane proteins from their natural environment typically results in structural and functional alterations and loss of function^48,50–53^, necessitating the use of membrane-mimetic systems to maintain protein solubility, stability, and activity^47,54^.

The aim of our study was to identify optimal conditions for the gentle, activity-preserving extraction of TRPC3 by retaining the protein’s native environment. To achieve this, we tested different nanodisc-forming polymers, including diisobutylene/maleic acid copolymer (DIBMA) and its derivative Carboxy-DIBMA, as well as dodecyl diglucoside (DDDG). DDDG is a non-ionic small-molecule amphiphile with a diglucose polar headgroup and dodecyl chain and is able to form lipid-bilayer nanodiscs in a similar way as the above polymers. In addition, we used different types of detergents for comparison, including the non-ionic detergents *n*-dodecyl-β-D-maltoside (DDM) and lauryl maltose neopentyl glycol (LMNG) and the zwitterionic detergent *n*-dodecyl-phosphocholine (FOS-choline). We investigated adherent HEK293 cells, *Komagataella phaffii (K. phaffii)* yeast cells, and Expi293F cells. Our results showed that Expi293F cells are the most suitable expression system for TRPC3, with DDDG and DDM exhibiting the most efficient extraction properties. Furthermore, we demonstrated that purified TRPC3 retains essential plasma membrane (PM) lipids and preserves its functionality, exhibiting no significant differences in channel activity compared to TRPC3 overexpressed in adherent HEK293 cells.

## Results

### Fusion of YFP and 3x Flag tags has no significant impact on TRPC3 activity

**Fig. 1:**
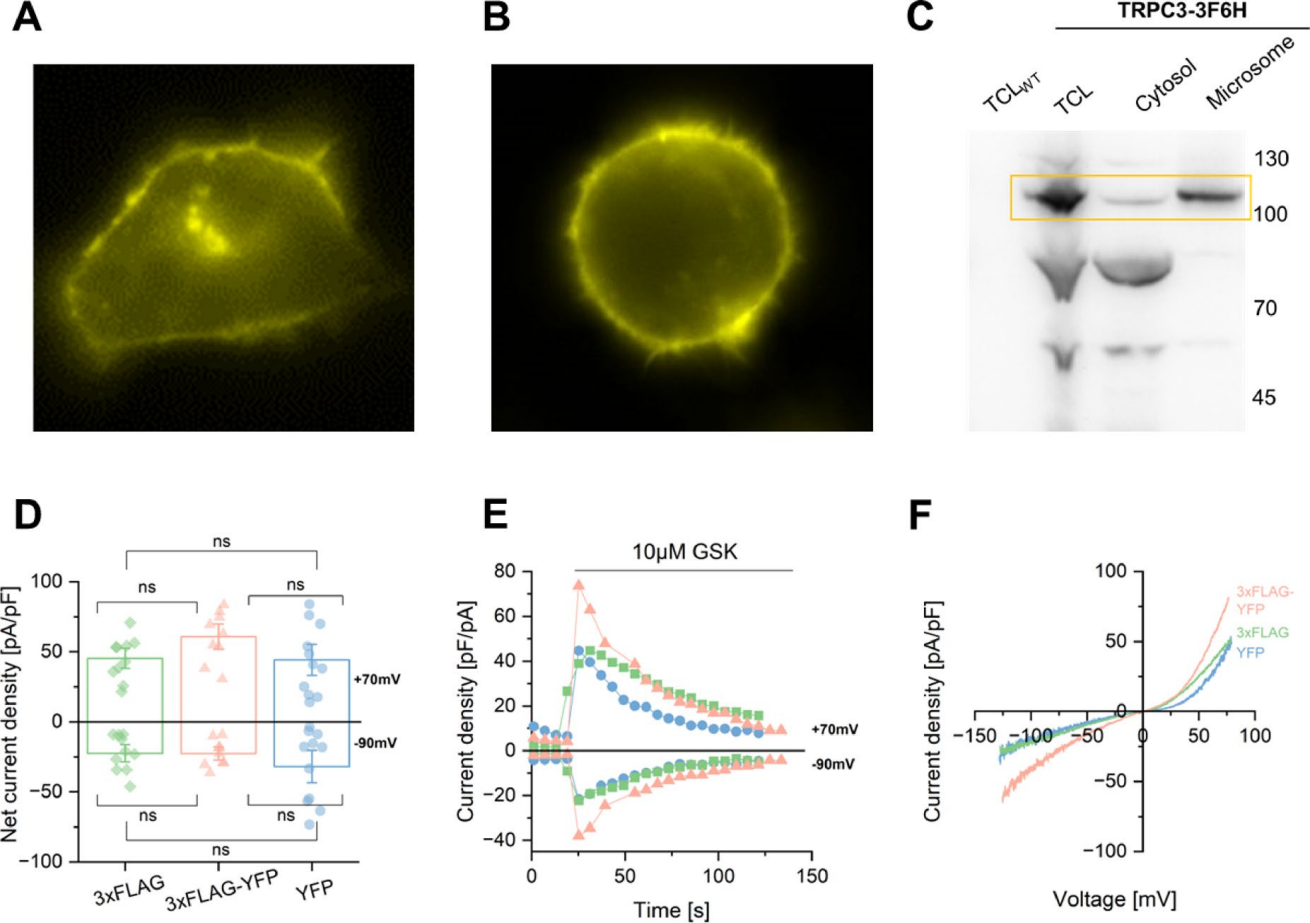
**Overexpression of TRPC3 in adherent HEK293 (A)**,** Expi293F (B)**,** and** ***K. phaffii*** **cells (C)**,** and electrophysiology of TRPC3-YFP-3xFLAG in adherent HEK293 cells by whole-cell analysis (D–F). A–C)** In all cell types, TRPC3 is prominently expressed in the PM. Overexpression of TRPC3 in HEK293 (**A**) and Expi293F cells (**B**) was examined by fluorescence microscopy, where the YFP tag was excited at 495 nm, and emission was detected at 530 nm. TRPC3 is highlighted in yellow localizing in the PM. **C)** TRPC3 is enriched in microsomal fractions of *K. phaffii*. A CBS7435 *his4*Δ strain and an otherwise isogenic strain expressing TRPC3-3F6H driven by the strong *AOX1* promoter were induced with methanol. Different fractions (total cell lysates (TCL)), as well as cytosolic and microsomal fractions) were prepared, separated by SDS–PAGE, and analyzed by immunoblotting with anti-FLAG antibody (TLC_WT_ = total cell lysate of *K. phaffi* without TRPC3 (negative controle), TLC = total cell lysate of *K. phaffi* expressing TRPC3). **D-F)** Whole-cell patch clamp analysis was conducted in adherent HEK293 cells activated with 10 µM GSK. The TRPC3–YFP–3 x FLAG construct (orange, *n* = 9), TRPC3–YFP construct (blue, *n* = 11), and TRPC3–3xFLAG construct (green, *n* = 10) were compared. No significant variances in TRPC3 channel activity were observed across the constructs. **D)** Statistical evaluation of maximum current densities of the TRPC3 channels at − 90/70 mV with mean ± SEM are displayed. Statistical analysis was performed using a two-tailed Student’s *t*-test for normally distributed data and a Wilcoxon test for non-normally distributed data, with significance denoted as follows: ns = non-significant, * *p* < 0.05, ** *p* < 0.01, *** *p* < 0.001. **E)** Representative time courses of current activation by 10 µM GSK in TRPC3 channels are shown. **F)** Representative *I*–*V* curves of activated TRPC3 channels with 10 µM GSK are displayed. Please note: Uncropped fluorescent images of TRPC3-YFP-3xFLAG over expression in adherent HEK293 and Expi293F cells are provided in the Supplementary Information (see Figure S1).

To validate overexpression and confirm the cellular localization of TRPC3 in adherent HEK293 and Expi293F cells, we transiently transfected both cell types with a pEYFP–3 x FLAG–TEV–TRPC3–C1 plasmid and performed fluorescence microscopy experiments. The YFP tag of the construct was excited at 495 nm, with emission detected at 530 nm. Both cell types showed TRPC3 overexpression localized to the PM (Fig. 1A **and B**). Due to the absence of the fluorescent tag in the construct for protein overexpression in yeast, we conducted a Western Blot analysis to assess TRPC3 localisation in *Komagataella phaffii* (syn. *Pichia pastoris*). Our results confirmed the presence of TRPC3 in the microsome of *K. phaffii*, as illustrated in Fig. 1C.

To evaluate if the activity of the TRPC3–3 x FLAG–YFP (3xFLAG-YFP) fusion protein, whole-cell voltage-clamp experiments were conducted using transiently transfected adherent HEK293 cells. As control, we used cells transfected with a TRPC3–YFP fusion (YFP) or a plasmid encoding TRPC3–3 x FLAG co-expressed with the empty pEYFP vector (3xFLAG). We compared the activity of the TRPC3 under these conditions to ensure that the fusion of TRPC3 with YFP along with 3 x FLAG tags does not interfere with channel functionality. Electrophysiological recordings demonstrated that the peak current densities (Fig. 1D **and E**) and current–voltage (*I*–*V*) relationships (Fig. 1F) of the 3xFLAG–YFP channel, activated by GSK1702934A (GSK^55^; 10 µM), were similar to those of YFP or 3xFLAG constructs. These results demonstrated that the 3xFLAG-YFP construct was suitable for further experiments.

### DIBMA and DDDG exhibit similar TRPC3 solubilization efficiencies compared to DDM

**Fig. 2:**
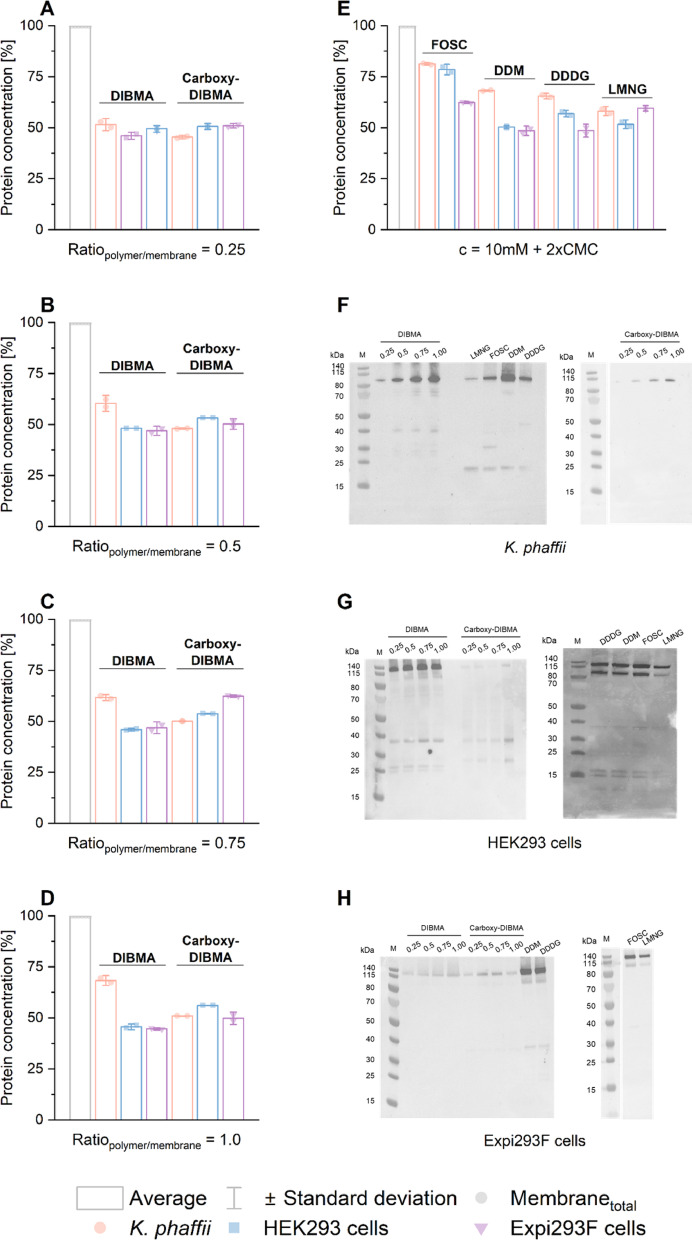
**BCA assay and Western Blot analysis was conducted on supernatant fractions containing all membrane proteins extracted with DIBMA**,** Carboxy-DIBMA**,** DDDG**,** DDM**,** l LMNG or FOS-Choline (FOSC) from adherent HEK293 (blue)**, ***K. phaffii*** **(orange)**,** and Expi293F suspension cells (purple). A-E)** Yields of extracted membrane proteins for DIBMA and Carboxy-DIBMA at different polymer/membrane mass ratios: *R* = 0.25 (**A**), *R* = 0.5 (**B**), *R* = 0.75 (**C**), and *R* = 1.0 (**D**). **E)** Yields of extracted membrane proteins for DDDG, DDM, LMNG and FOS-Choline (FOSC). Except FOS-choline, which resulted in higher protein yields, and membrane-protein extraction from *K. phaffii*, all extraction agents yielded similar protein yields (50–60%) from all three expression systems. The membrane-protein concentration was normalized to the total membrane-protein concentration before solubilization and expressed as a percentage yield (%). **F-G)** Western Blot analysis was conducted on the supernatant fractions extracted from adherent *K. phaffii* (**F**), adherent HEK293 cells (**G**) *and* Expi293F (**H**) cell membranes using DIBMA and Carboxy-DIBMA at various polymer-to-membrane ratios ranging from *R* = 0.25 to 1.0, as well as DDM, DDDG, FOS-Choline (FOSC), and LMNG. A specific TRPC3 antibody was used to target TRPC3. **F**) DIBMA at *R* = 1.0 and DDM extract the highest quantity of TRPC3. **G**) DIBMA extracts the highest quantity of TRPC3. **H**) DDM and DDDG extract the highest amount of TRPC3. **Note**: Please note that the Western blots on the right side of Panels F and H were cropped from the same blot. For the full, uncropped Western blot, refer to Supplementary Information, Figure S6.

To determine the most effective expression system and extraction agent for TRPC3, we overexpressed TRPC3 in adherent HEK293 cells, *K. phaffii* cells, and Expi293F cells. Next, we extracted membrane proteins, including TRPC3, using two nanodisc-forming polymers (DIBMA and Carboxy-DIBMA)^56,57^, a nanodisc-forming small-molecule amphiphile (DDDG)^48,58^, and three commonly used detergents (DDM, LMNG, and FOS-choline), all at a constant membrane concentration of 25 mg/mL. We analyzed the supernatant and pellet fractions through SDS-PAGE (**see SI Figure S2 and S4**) and conducted BCA assay analysis (Fig. 2, **SI Figure S5**) to assess the overall membrane-protein concentration and the extraction efficiency of each compound.

Both amphiphilic copolymers, DIBMA and Carboxy-DIBMA, achieved good overall membrane-protein yields (40–50%) across all three expression systems, with a slightly higher yield (~ 60%) observed for DIBMA using *K. phaffii* cells. In contrast, detergent performance varied considerably: while all detergents resulted in high membrane-protein yields from *K. phaffii* cells (70–80%), yields from adherent HEK293 cells and Expi293F suspension cells were similar (50–60%) (Fig. 2A–D). Overall, protein yields extracted with DDDG, DDM, and LMNG from HEK293 and Expi293F cells were similar to those achieved with DIBMA and Carboxy-DIBMA. As expected, the harsh, zwitterionic detergent FOS-choline resulted in higher protein yields (60–80%) across all three expression systems (Fig. 2E).

Recognizing the limitations of the BCA assay in specifically quantifying TRPC3 extraction, we conducted a Western Blot analysis using a TRPC3-specific antibody (Fig. 2) to determine which compound extracted the highest quantity of TRPC3. Our results showed that different extraction agents were optimal for different expression systems. Specifically, (I) for adherent HEK293 cells (Fig. 2G), DIBMA was superior in extracting the highest amount of TRPC3; (II) for *K. phaffii* (Fig. 2F), both DIBMA and DDM were effective in extracting the largest amounts of TRPC3; (III) in Expi293F cells (Fig. 2H), DDDG and DDM exhibited the highest extraction efficiencies for channel.

### DDDG and DDM are suitable for TRPC3 purification under native conditions

**Fig. 3:**
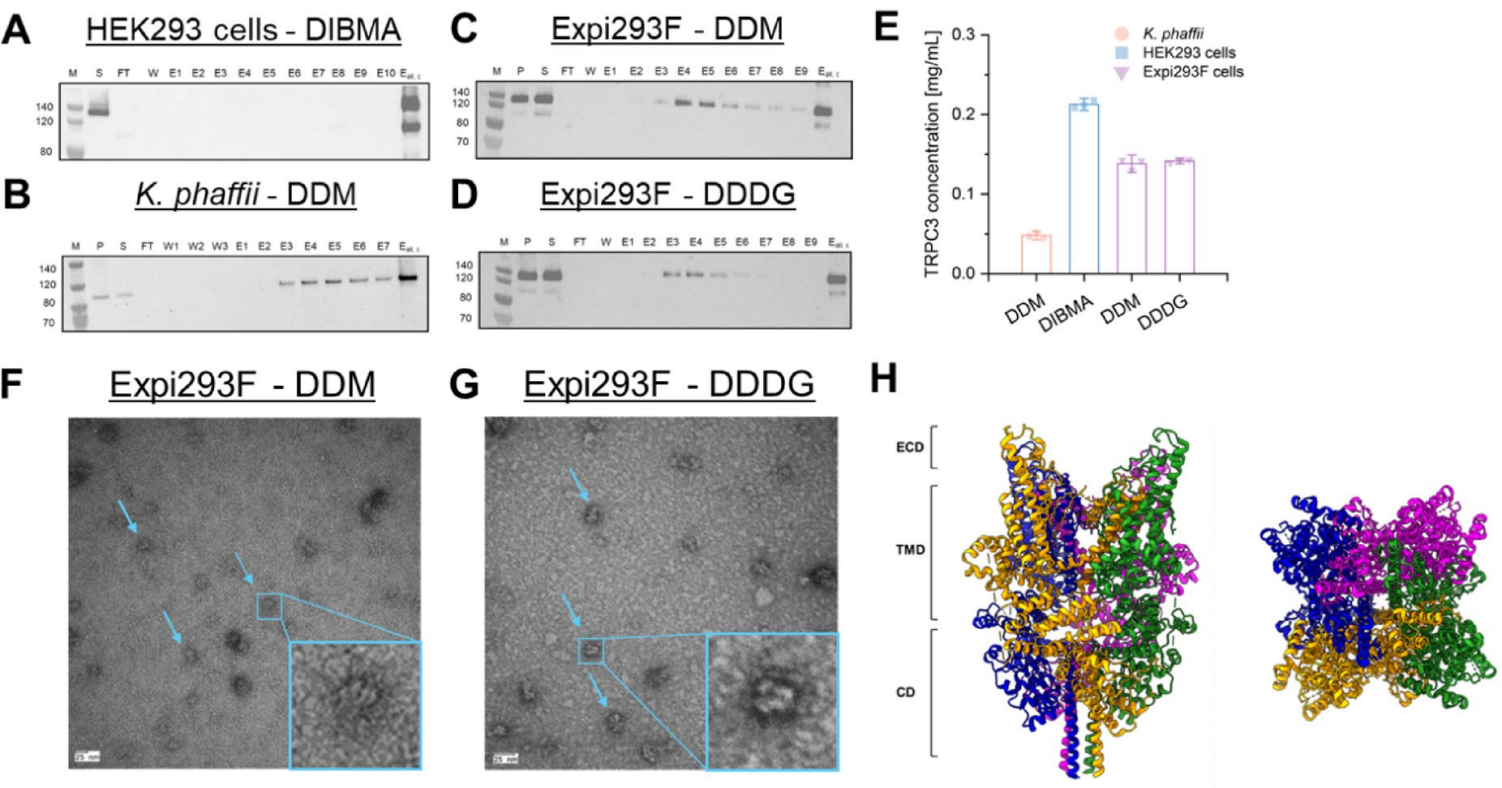
Western blot analysis and negative-stain electron microscopy of purified TRPC3. TRPC3 was purified from adherent HEK293 cell membranes using DIBMA (A), *K. phaffi* cell membranes using DDM (*n* = 3, B), and Expi293F cell membranes using DDDG (*n* = 6, C) or DDM (*n* = 6, D). The resulting concentrations of purified TRPC3 are shown in (**E**). TRPC3 protein concentration (V_total_ = 150 µL) was lower from *K. phaffi* cell membranes compared to adherent HEK293 or Expi293F cells. Specifically, TRPC3 concentration was 0.05 mg/mL from *K. phaffii* cell membranes using DDM, 0.2 mg/mL from HEK293 cell membranes using DIBMA, and 0.15 mg/mL from Expi293F cells using either DDM or DDDG. Negative-stain electron microscopy images of TRPC3 extracted with DDM (**F**) or DDDG (**G**) from Expi293F cells reveal the tetrameric structure of TRPC3 channels (blue arrows). The blue box highlights the magnification of a single TRPC3 tetramer as observed in the electron microscopy images. **H**) General cryo-EM structure of the closed homomeric TRPC3 channel from the side (left) and top (right) view (PDB: 7DXB)^42^. **Note**: Please note that the Western blots in Panel A-D were cropped. For the full, uncropped Western blot, refer to Supplementary Information, Figure S7.

To purify TRPC3, we solubilized 25 mg of the membrane fraction from HEK293 cells with DIBMA (Fig. 3A, **SI Figure S7A**), 200 mg of the membrane fraction from *K. phaffii* with DDM (Fig. 3B, **SI Figure S7B**), and 25 mg of the membrane fraction from Expi293F cells with either DDM (Fig. 3C, **SI Figure S7C**) or DDDG (Fig. 3D, **SI Figure S7D**). Due to the unstable overexpression of TRPC3 in *K. phaffii*, a higher amount of the membrane fraction was required compared to the amounts used for HEK293 or Expi293F cells. Successful purification was achieved from each expression system (Fig. 3).

TRPC3 was purified from adherent HEK293 cells with DIBMA at a final concentration of 0.2 mg/mL. For Expi293F cells, TRPC3 was purified with DDM or DDDG at a concentration of approximately 0.15 mg/mL from the same amount of membrane fraction (25 mg). However, the total TRPC3 protein concentration from *K. phaffii* was only 0.05 mg/mL (Fig. 3E). Despite the efficient extraction of TRPC3 with DIBMA from adherent HEK293 cells, this process proved more time-consuming and material-intensive compared to using Expi293F cells, hence we decided to only continue with Expi293F cells, as well as DDM and DDDG as extraction agents.

As the following step, our objective was to verify the native homotetrameric structure of TRPC3 post-purification. To accomplish this, we performed negative-stain electron microsopy (nsEM) (Fig. 3F and G). Our findings demonstrated that TRPC3 was purified under native conditions, as manifested by the preservation of its tetrameric structure with both extraction agents (Fig. 3F and G). This observation aligns well with the previously reported TRPC3 cryo-EM structures^15,16,42,59^. As illustrated in the Fig. 3H, the four monomers clearly form a flower-shaped structure. This configuration is also evident in the negative stain electron microscopy images shown in the Fig. 3F and G.

To validate the extraction of TRPC3 under native-like conditions using both DDM and DDDG, we performed lipidomic analysis via mass spectrometry on purified TRPC3 samples. As anticipated, a variety of native lipid species were detected in both preparations, including diacylglycerol (DAG), phosphatidylcholine (PC), and phosphatidylethanolamine (PE) (**SI Figure S8**), indicating that essential components of the surrounding lipid environment were retained during the purification process. Unexpectedly, we also observed substantial amounts of triglyceride (TG) species co-extracted with TRPC3 in both detergent systems (**SI Figure S8**). Given that triglycerides are not typically present in significant concentrations within the PM, we hypothesize that their presence is likely an artifact of the solubilization process. This suggests that these neutral storage lipids may have been non-specifically incorporated into the detergent micelles or co-purified from intracellular lipid droplets. Nevertheless, the consistent co-extraction of triglycerides across both detergent conditions presents an intriguing observation that may reflect previously unrecognized lipid-protein associations or compartmental cross-contamination. While their physiological relevance to TRPC3 function remains uncertain, this finding provides a valuable starting point for further exploration into how non-canonical lipids may influence membrane protein purification and stability.

For the main purpose of our study—upscaling TRPC3 expression for future cryo-EM investigations—, we decided to proceed with Expi293F cells and use DDDG and DDM as the most suitable for protein extraction from this expression system (Fig. 2H **and** Fig. 3).

### Purified and reconstituted TRPC3 exhibits native-like single-channel features

**Fig. 4:**
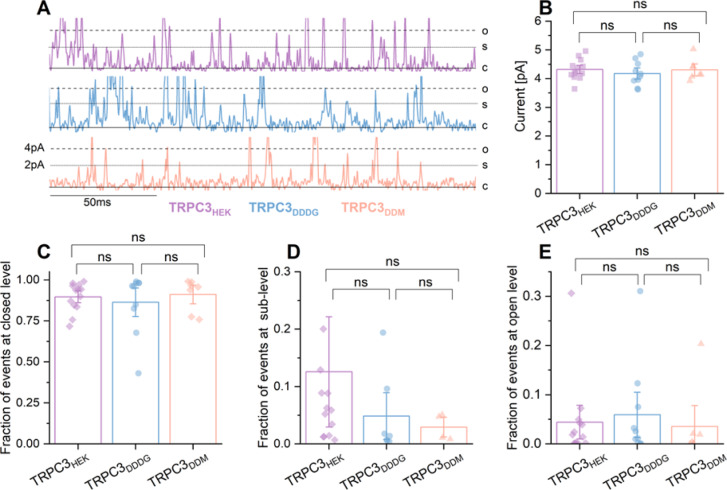
**Single-channel patch-clamp experiments were conducted on TRPC3 in HEK293 cells (purple) and on reconstituted TRPC3 extracted from Expi293F cells using DDDG (blue) or DDM (orange). (A)** Representative single-channel currents showing 200 ms of GSK-induced TRPC3 activity. TRPC3 was either expressed in HEK293 cells (purple) or purified from Exppi293F cells using DDDG (blue) or DDM (orange) and reconstituted into DPhPC bilayers. GSK was applied at 10 µM for adherent HEK293 cells and 20 µM for reconstituted TRPC3. Currents were recorded at + 80 mV. Channel states are indicated as closed (c), sublevels (s), or open (o). **(B)** Unitary currents at the open (o) level in response to repeated GSK applications of (10 µM for adherent HEK293 cells and 20 µM for reconstituted TRPC3) at a membrane potential of + 80 mV. Data are presented as means ± SEM. Statistical significance was determined using a two-tailed multiple *t*-test for normally distributed data and the Wilcoxon test for non-normally distributed data. Non-significant differences are denoted as “ns.” **C–E)** Fraction of events corresponding to the closed (panel **C**), sublevel (panel **D**), and open (Panel **E**) states, derived from Gaussian fitting of single-channel recordings from HEK293 cells expressing TRPC3 (purple), reconstituted TRPC3 purified with DDDG (blue) or with DDM (orange). GSK was applied at 10 µM for HEK293 cells and 20 µM for reconstituted TRPC3 at a membrane potential of + 80 mV. Data are presented as means ± SEM. Statistical significance was assessed using a two-tailed multiple *t*-test or the Wilcoxon test, with differences considered significant at * *p* < 0.05, ** *p* < 0.01, and *** *p* < 0.001. Non-significant differences are indicated as “ns”. We demonstrated that purified TRPC3 remains functional and its activity is comparable to TRPC3 overexpressed in HEK293 cells. **Please note**: Uncropped representative traces from Fig. 4A are shown in the Supplementary Information including the statistical analysis of the fraction of events at multiple levels (**SI Figure S12**).

To validate the functionality of TRPC3 purified from Expi293F cells using either DDDG or DDM (**SI Figure S9**), we reconstituted the purified TRPC3 channels into a synthetic lipid bilayer composed of 5 mg/mL 1,2-diphytanoyl-*sn*-glycero-3-phosphocholine (DPhPC) as it ws previously done in Garten et al., 2015^62^. We then evaluated channel activity by applying voltages at + 80 mV and stimulating with the direct activator GSK1702934A (GSK, 20 µM)^63^. Moreover, we conducted single-channel patch-clamp recordings using TRPC3–YFP–3 x FLAG overexpressed in HEK293 cells, following the method described by Clarke et al. 2024^64^. The single-channel analysis showed similar GSK-induced TRPC3 activity in HEK293 cells (Fig. 4A) and in a DPhPC bilayer (Fig. 4A). Electrophysiological analysis revealed no significant differences in comparison of GSK (20 µM)-stimulated TRPC3 channel activity recorded from heterologously expressed TRPC3 in HEK293 and from reconstituted TRPC3 purified with DDDG or DDM (Fig. 4). All TRPC3 channels exhibited similar unitary conductance with *y*_TRPC3/HEK_ = (54.0 ± 1.2) pS, *y*_TRPC3/DDDG_ = (52.2 ± 1.6) pS, and *y*_TRPC3/DDM_ = (53.8 ± 1.7) pS (Fig. 4B).

Furthermore, the TRPC3 in HEK293 cells and TRPC3 channels purified with DDDG or DDM and reconstituted into a DPhPC bilayer showed similar gating behavior as those characterized by standard patch-chlamp recordings in HEK293 cells. Importantly, reconstituted TRPC3 also predominantly remained in a long-lived closed conformation, very similar to TRPC3 overexpressed in HEK293 cells (Fig. 4C). We measured similar gating transitions for TRPC3, including the fully open state and the sub-level state, indicating indistinguishable functional activities for all conditions (Fig. 4D **and E**). These findings demonstrate that TRPC3 retained its functionality post-purification and that were no significant differences in TRPC3 channel activity upon GSK stimulation between reconstituted TRPC3 and TRPC3 overexpressed in HEK293 cells. Control experiments using DMSO, GSK, or detergents, all conducted in the absence of reconstituted TRPC3, showed no impact on the stability of the lipid bilayer (**SI Figure S10**), confirming that the GSK-induced activity was attributable to the presence of TRPC3. To further verify that the observed channel activity was indeed due to reconstituted TRPC3, we inhibited TRPC3 post-activation with the selective inhibitor Pyr10^65^, which completely suppressed TRPC3 acitity in both HEK293 cells and reconstituted channels (**SI Figure S11**).

## Discussion

Despite the increasing number of high-resolution structures of TRPC3, all studies to date have captured the channel exclusively in its closed conformation—even under activating conditions using direct agonists such as diacylglycerol (DAG)^15,16,42^. We hypothesized that the inability to capture the open conformation of TRPC3 could stem from two specific characteristics of the channel: (1) its strong dependence on its lipid environment, including cholesterol^14,61^, DAG^12^, and PIP_2_^45,66,67^; and (2) the exceptionally short-lived channel open state, typically resulting in characteristic open times around or below 1 ms^8,12,68^ which may be affected by changes in lipid composition during protein purification leading to a further reduced open state dwell time. Therefore, we aimed to determine the possible hurdles occurring during protein extraction that could likely lead to a non-functional TRPC3 channel during cryo-EM.

Cryo-EM structure determination involves extracting proteins from their native lipid environment, which can disrupt important lipid interactions and result in a closed-pore conformation, even in the presence of agonists. Detergents and, more recently, nanodisc-forming amphiphiles are frequently used to extract membrane proteins while attempting to preserve their native properties^47,51^. Commonly used detergents include non-ionic alkyl glycosides like maltosides and glucosides^51–53,69–72^, with longer-chain derivatives (C10–C12) typically being more gentle than their shorter-chain counterparts (C7–C9)^47,51,53^. Notably, DDM is often seen as the “gold standard” due to its efficient solubilization and rather gentle, activity-preserving properties for many membrane proteins^51^. Amphiphilic polymers such as DIBMA^56,57,73^ and styrene/maleic acid (SMA)^73–75^ copolymers and, more recently, derivatives such as Glyco-DIBMA^76^ and Sulfo-DIBMA^77^, extract membrane proteins into native nanodiscs that encapsulate both proteins and lipids and a native-like bilayer environment^73,78^. Recently, similar observations have been made for small-molecule amphiphiles such as DDDG and its fluorinated octyl diglucoside counterpart F_6_ODG^47–49,58^.

TRPC3 has previously been purified using two non-ionic detergents, digitonin^15^ and LMNG^16,42^, for structural analysis. These two detergents are generally classified as gentle detergents due to their longer chain lengths and mild solubilization properties^53^. However, this mildness comes at a cost: both digitonin and LMNG typically show low extraction efficiency—a common limitation among gentle detergents^51,53,79^. Consequently, although these detergents are advantageous for preserving protein–lipid interactions^79^they often solubilize only limited amounts of the target protein, potentially yielding insufficient material for structural and functional analysis, particularly in applications requiring high quantities of functional protein, such as cryo-EM.

Given that cryo-EM requires a substantial amount of functional TRPC3, we expanded our detergent screening beyond digitonin and LMNG to include DDM, FOS-choline, and DDDG, alongside amphiphilic polymers, to identify extraction agents capable of isolating TRPC3 in sufficient quantities for subsequent functional and structural analysis.

Furthermore, TRPC3 was previously purified from adherent HEK293 cells^80^ or HEK293 suspension cells, which required time and viral transfection for efficient DNA delivery^15,16,42^. The primary aim of our study was to identify the most suitable expression system for isolating TRPC3 in large quantities while preserving its native lipid environment for structural analysis. To achieve this, we tested three different expression systems: yeast *K. phaffii*, adherent HEK293, and suspension Expi293F cells. Additionally, we evaluated six different extraction agents: DIBMA, Carboxy-DIBMA, DDDG, DDM, LMNG, and FOS-choline. Our study demonstrates, for the first time, the efficiency of *K. phaffii* and Expi293F cells as alternative expression systems for large-scale TRPC3 protein production (Fig. 1A-C). Moreover, Expi293F cells enabled the production of large quantities of the protein without the need for viral transfection.

To identify the optimal expression system and extraction compound which stabilizes TRPC3 during purification, we solubilized the membrane fractions using DIBMA, Carboxy-DIBMA, DDDG, and three detergents: LMNG, DDM, and FOS-Choline and perfromed Western blot and BCA-Assay analysis (Fig. 2, **SI Figure ****S1****−6**). Western Blot results showed varied effectiveness among all used compounds (Fig. 2), leading to the selection of DDM for *K. phaffii* cells, DIBMA for HEK293 cells, and DDM and DDDG for Expi293F cells for TRPC3 purification (Fig. 2, **SI Figure ****S1****−6**). Purified TRPC3 concentration from *K. phaffii* membranes (0.05 mg/mL, Fig. 3E) was four times lower than from HEK293 cells (0.2 mg/mL) and one-third of that from Expi293F cells (0.15 mg/mL) (Fig. 3A-E, **SI Figure S7**). These results indicate that *K. phaffii* may not be the optimal expression system for TRPC3 due to its low yield during purification. Additionally, TRPC3 is a cholesterol-dependent channel, while *K. phaffii* does not produce cholesterol but rather ergosterol^81,82^. Consequently, we excluded *K. phaffii* from further experiments. Despite the promising extraction of TRPC3 from HEK293 cells with DIBMA, the process was more time-consuming and material-intensive compared to Expi293F cells, which require smaller amounts of material and grew rapidly. Therefore, we excluded HEK293 cells as well and continued experiments only with Expi293F cells. Finally, negative-stain electron microscopy images revealed that the tetrameric structure of TRPC3 purified from Expi293F cells with DDDG or DDM was successfully preserved (Fig. 3F **and G**). Overall, our results indicate that among all expression systems and extraction agents tested, Expi293F cells combined with either DDDG or DDM provide the most favorable conditions for high-yield overexpression, extraction, and purification of TRPC3. Therefore, subsequent experiments were conducted exclusively under these optimized conditions to support downstream structural and functional studies.

The first hypothesis of our study was that TRPC3 retains essential lipid interactions necessary for maintaining a stabilized conformation during solubilization and purification. As a nano-disc forming agent, DDDG can co-extract lipids and form lipid-bilayer nanodiscs^48^. A similar preservation of lipid interactions might also occur with DDM, even though this mild detergent forms mixed micelles rather than lipid-bilayer nanodiscs^51^. In this case, incomplete delipidation upon DDDG- and DDM-mediated solubilization could still allow TRPC3 to retain its essential lipid partners during purification, resulting in a structural stabilized channel during purification (Fig. 3F **and G**). To determine whether TRPC3 retains its most essential lipids during purification, we purified the channel using either DDM or DDDG and conducted mass spectrometry analysis to identify which lipids, if any, remain bound. Mass spectrometry analysis revealed that several native lipids, including DAG, PC, and PE, co-purify with TRPC3 when extracted with DDM or DDDG (**SI Figure S8**). We note that only DAG has a well-established functional role in TRPC3 activation, whereas the contributions of PC and PE remain less clear. Their consistent presence suggests that detergent extraction allow partial retention of the native lipid environment (**SI Figure S 8**) which could potentially stabilize TRPC3 channels during purification.However, we recognize that co-purification alone does not establish functional relevance, and further lipid reconstitution experiments will be required to test how these lipids contribute to gating. Interestingly, mass spectrometry analysis revealed the absence of a key cholesterol molecule^61^ in both purified TRPC3 samples (**SI Figure S8)**. This is likely attributable to the mild, non-ionic nature of both extraction compounds, which are known to poorly solubilize lipid rafts—cholesterol- and sphingolipid-rich microdomains within the plasma membrane. Previous studies have demonstrated that similar non-ionic detergents, such as Triton X-100, are inefficient in disrupting these ordered domains due to their detergent-resistant properties^60^. Given that cholesterol has been implicated in modulating TRPC3 channel function^61^its exclusion from the detergent micelles raises concerns about the completeness of the native lipid environment in current preparations. In light of these findings, further optimization of the solubilization protocol—potentially through the inclusion of cholesterol analogs such as cholesteryl hemisuccinate, or alternative detergents capable of disrupting raft domains—may be necessary to preserve physiologically relevant lipid-protein interactions critical for TRPC3 function and structure.

The second obstacle to capturing the open state of TRPC3 in structural studies may lie in the channel’s gating behavior, which might be highly dependent on the presence of a specific lipid environment^45,61^. Single-channel data suggest that the open state of TRPC3 is relatively short-lived, with estimated dwell times around 0.13 ms^68^, 0.2 ms^12^, and 0.28 ms^8^, depending on the activator. Extraction of the TRPC3 channel from its native lipid environment can significantly alter its gating behavior. Consequently, in the second part of our study, we aimed to investigate the activity of purified TRPC3 (Fig. 4, **SI Figure S10-12**). Post-purification analysis showed no significant differences in the functionality of purified and reconstituted TRPC3 compared to TRPC3 overexpressed in HEK293 cells (Fig. 4). We compared the single-channel activity of TRPC3 overexpressed in HEK293 cells with that of TRPC3 reconstituted in a lipid bilayer after purification with DDDG or DDM. Surprisingly, although the synthetic bilayer was composed solely of DPhPC, we observed no significant differences in the gating behavior of reconstituted TRPC3 compared to TRPC3 overexpressed in HEK293 cells (Fig. 4). Finally, we demonstrated that Pyr10 inhibits the activity of TRPC3 overexpressed in HEK293 cells as well as reconstituted TRPC3 in an artificial lipid bilayer (**SI Figure S11**). Our electrophysiological experiments demonstrated that TRPC3 purified with DDDG or DDM and reconstituted into a synthetic lipid bilayer exhibited comparable gating behavior and unitary conductance to overexpressed TRPC3 in HEK293 cells.

These findings confirm that a fraction of TRPC3 remains structurally stabilized during solubilization and purification, enabling its successful reconstitution into a synthetic lipid bilayer. There the channel exhibit channel activity comparable to that observed in HEK cells (Fig. 4). Notably, Nikolaev et al.^83^also observed no significant differences in the dwell time of TRPC6 reconstituted in a lipid bilayer composed of phosphatidylcholine from soybean compared to overexpressed TRPC6 in HEK293 cells ( = 1 ms and = 1.9 ms, respectively)^83^. To further dissect the lipid specificity of TRPC3 gating and stabilize its open conformation for structural analysis, future reconstitution experiments should incorporate more complex lipid mixtures—including cholesterol, DAG, and PIP₂—known to modulate channel activation. Such conditions would provide a more physiologically relevant environment and increase the probability of capturing the channel in an open conformation suitable for cryo-EM.

Overall, our results identify optimal conditions for TRPC3 expression, solubilization, and purification using DDM and DDDG, under which a fraction of the channels remains structurally stabilized for downstream functional studies. We further demonstrate that these stabilized TRPC3 fractions can be successfully reconstituted into a synthetic lipid bilayer, where they exhibit channel activity comparable to that observed in HEK cells.Although our initial hypotheses were only partially validated, we identified key factors—such as the absence of cholesterol and the consistent co-purification of DAG, PC, and PE—that will guide future optimization of TRPC3 purification for structural analysis. When further refined, these insights may enable structural capture of the channel in its open state and deepen our understanding of TRPC3’s lipid-dependent gating mechanisms.

## Methods

### Cloning of constructs

The coding sequence of human *TRPC3* (Uniprot database ID: Q13507-3) was cloned alone or together with a 3 x FLAG tag into the pEYFPpeYFP–C1 vector (Clontech, Saint-Germain-en-Laye, France). Furthermore, human TRPC3 and 3 x FLAG tag was cloned into pEEmptypeEmpty-C1 vector.

### Plasmid DNA Preparation

DNA preparations were performed using a QIAGEN Plasmid Maxi Kit. *Escherichia coli* carrying the pEYFP–3 x FLAG–TEV–TRPC3–C1 vector, pEYFP–TRPC3–C1 vector or pEEmpty-TRPC3–3 x FLAG–C1 vector was cultured in 300 mL LB Medium overnight and harvested the following day. The extraction of plasmid DNA was performed following the manufacturer’s protocol.

### Expression of TRPC3 in HEK293 cells

Human embryonic kidney 293 (HEK293) cells (Cell Lines Service, product number: 300192) were cultured in Dulbecco’s Modified Eagle Medium (DMEM, D6429, Invitrogen) supplemented with 10% fetal bovine serum (FBS), 1% streptomycin, 1% penicillin, 1% L-glutamine, and 1% HEPES at a constant temperature of 37 °C and under an atmosphere of 5% CO_2_. For plasmid transfection in electrophysiology studies, the culture media was aspirated, and HEK293 cells were rinsed with PBS. Cells were then incubated with accutase (500 µL) for 5 min at 37 °C. The detached cell suspension was mixed with fresh DMEM in a 2:1 ratio (DMEM: Accutase). A suspension of 1 × 10^5^ cells was centrifuged at 600 rpm for 4 min. The supernatant was discarded, and the cell pellet was suspended in Opti-MEM I serum-free medium (60 µL). Cells were transiently transfected with 1 µg plasmid DNA using PolyJet (SignaGen Laboratories) according to the manufacturer’s protocol. Cells were seeded on 6 × 6 mm glass coverslips, and the medium was changed after 8 h of incubation. Experiments were performed 20–24 h after transfection.

For microscopy experiments, cells were transiently transfected with 1 µg plasmid DNA using polyethyleneimine (PEI) with a DNA/PEI ratio of 1:3. Cells were grown on 3 × 3 cm glass coverslips until reaching 60% confluency. The medium was exchanged for an antibiotic-free medium containing only 2.5% FBS, 1% L-glutamine, and 1% HEPES. DNA and PEI were mixed in 60 µL OptiMEM and added to the cells. Following a 36 h incubation, TRPC3 overexpression was verified using a fluorescence microscope.

For protein purification, cells were grown in 50 × 15 cm dishes until reaching 80% confluency. Then, the medium was exchanged for an antibiotic-free medium containing only 2.5% FBS, 1% L-glutamine, and 1% HEPES. Cells were transiently transfected with 1 µg plasmid DNA using polyethyleneimine (PEI) at a DNA-to-PEI ratio of 1:3, mixed in OptiMEM. After 36 h of incubation, cells were harvested using a cell scraper and centrifuged at 1 500 *g* and 4 °C for 15 min. Cell pellets were frozen at − 80 °C until further use in experiments.

### Expression of TRPC3 Expi293F cells

Expi293F cells were cultivated in 100 mL Expi293 Expression Medium (Gibco) under gentle shaking, at 37 °C and under an atmosphere of 5% CO_2_. Expi293F cells were transfected using 1 µg plasmid DNA/mL Medium with ExpiFectamine according to the manufacturer’s protocol. 20 h after transfection of cells the Enhancer solutions were added to increase TRPC3 protein expression. Cells were harvested 48 h later and TRPC3 overexpression was analyzed by fluorescence microcopy and Western Blot analysis.

### Fluorescence Microscopy Imaging

To assess TRPC3 expression in HEK293 and Expi293F cells, total internal reflection fluorescence microscopy (TIRFM) was employed. TIRF microscopy was conducted using an Observer D1 microscope (Zeiss, Jena, Germany) equipped with a Visitron TIRF system (Visitron Systems, Puchheim, Germany). Cells were observed through a 100 × oil immersion Zeiss TIRF objective, with YFP being excited by a 488 nm diode laser. Image acquisition utilized a Prime BSI Express sCMOS camera (Teledyne Photometrics, Tucson, USA) at 515 nm. Subsequent analysis of TRPC3 localization was performed using ImageJ.

### Cell membrane preparation from HEK293 and Expi293F cells

Cell membranes were obtained by resuspending cell pellets in lysis buffer containing 150 mM NaCl, 50 mM Tris, 0.1 M Sucrose, and 1 x cOmplete Protease Inhibitor (EDTA-free), followed by disruption using a glas homogenizer. The crude extract (CE) was then subjected to centrifugation at 1 500 *g* and 4 °C for 15 min. The resulting pellet (P1) was stored at − 20 °C, while the supernatant (S1) underwent further centrifugation at 20 000 *g* for 60 min at 4 °C. The resultant pellet (P2) represented the membrane fraction containing the overexpressed TRPC3, and the supernatant (S2) was preserved at − 20 °C. For adherent HEK293 cells, a membrane pellet weighing 450–500 mg was obtained from 50 × 15 cm dishes (2 L culture medium), whereas 1.4 g of membrane was obtained from a 100 mL culture of Expi293F cells. All further experiments were conducted at a membrane concentration of 25 mg/mL unless otherwise specified. To confirm successful cell membrane preparation, Western Blot analysis was performed as described in the section “SDS-PAGE and Western Blot”.

#### *Cloning and strain production ofK. phaffiioverexpressing pPpT4 - TRPC3–3F6H*

All PCRs were conducted utilizing Phusion DNA polymerase (Thermo Fisher Scientific Inc., St. Leon-Rot, Germany). The accuracy of all constructs was confirmed through nucleotide sequence analysis. A C-terminally tagged variant of the TRPC3 gene (TRPC3–3xFlag–His_6_/TRPC3–3F6H) was cloned into pPpT4^84^ using Gibson assembly^85^. The plasmid map of pPpT4 - TRPC3–3F6H is available as Supplementary Material. Expression cassettes were generated by linearization of pPpT4–TRPC3–3F6H using *Smi*I, followed by gel purification. Transformation of *K. phaffii* CBS7435 *his4*Δ^84^ was done according to the condensed protocol of Lin-Cereghino et al.^86^ After transformation and regeneration, cells were plated onto YPD plates (2% peptone, 1% yeast extract, 2% glucose, 2% agar) containing 100 µg/ml Zeocin. Integration of cassettes into the yeast genome was verified by cPCR, and the strain further used for production of TRPC3 (yLB226: CBS 7435 *his4*Δ P_*AOX1*_–TRPC3–3 x FLAG–His_6_:Zeo) was conserved as glycerol stock at − 80 °C.

#### *Cell cultivation*,* TRPC3 expression*,* and isolation of the microsomal fractions fromK. phaffii*

Strain yLB226 was grown in 500 mL of BMG media (1% glycerol, 13.4 g/L Yeast Nitrogen Base (without amino acids), 4*10 5% biotin, 100 mM K_2_HPO^4^/KH_2_PO^4^, pH 6) for 24 h. Gene expression was induced by the addition of 1% methanol, and 0.5% methanol was added every 12 h for 48 h. Cell disruptions and the preparation of microsomal fractions were done as described before^87^. Briefly, cells were harvested by centrifugation (5 000 rpm at 4 °C for 5 min), washed with ddH_2_O, and centrifuged again (5’000 rpm at 4 °C for 5 min). Cells were resuspended in 25 mL of pre-cooled (4°C) 20 mM TE-Buffer (20 mM Tris/HCl, 1 mM EDTA, pH = 8, 2 mM PMSF) and disrupted using glass beads. To remove cell debris, samples were centrifuged at 5 000 rpm and 4 °C for 10 min. Next, the resulting total cell lysate was centrifuged at 10’000 rpm and 4 °C for 15 min. This step was repeated. The supernatant was then ultracentrifuged at 45’000 rpm and 4 °C for 45 min. The resulting supernatant contains the cytosolic fraction, and the cell pellet the microsomal fraction. Microsomal pellets were resuspended in 1 mL of PBS buffer.

#### Immunoblot analysis of TRPC3-3F6H

All steps were performed at 4 °C. 10 µg of proteins from total cell lysates (TCL), and cytosolic fractions, as well as 2 µg of proteins from microsomal fractions were precipitated by the addition of 600 µL of 25% trichloroacetic acid (TCA) for 1 h. Afterwards, samples were centrifuged for 10 min at 10’000 rpm. After discarding the supernatant, pellets were washed with 1 ml of ice cold ddH_2_O and resuspended in 50 µl of NuPAGE™ sample buffer. Samples were centrifuged at 10’000 rpm for 20 s, and 12 µl of the supernatant loaded onto NuPAGE Mini Protein Gels (12%, Bis-Tris, 1.0 mm; Thermo Fisher Scientific Inc), and resolved at 200 V. Electrophoretical transfer of the proteins to a nitrocellulose membrane was done using a wet transfer apparatus (NuPAGE™, Thermo Fisher Scientific Inc). For blocking, membranes were incubated for 1 h in TBST-BSA (5%) at room temperature. After blocking, the membranes were incubated overnight at 4 °C in TBST-BSA (2.5%) with mouse ANTI-FLAG^®^ M2-Peroxidase (HRP) antibody (1:2 500; Sigma Aldrich) and then washed with TBS-T (three times with ≥ 10 mL). Enhanced chemiluminescent signal detection (Clarity Max Western ECL Substrate, Bio-Rad) was used to visualize immunoreactive bands. PageRuler pre-stained protein ladder (Thermo Scientific™) was used as molecular weight marker.

#### *Extraction of TRPC3 fromK. phaffii*, *HEK293*,* and Expi293F cell membranes*

Cell membranes were first resuspended in 150 mM NaCl, 50 mM Tris and 10% glycerol at a final concentration of 25 mg/mL. For solubilization, dodecyldiglucoside (DDDG), FOS-Choline, lauryl maltose neopentyl glycol (LMNG), and the traditional detergent dodecyl-β-D-maltoside (DDM) were used at constant concentrations of 10 mM plus twice the critical micellar concentration (CMC). Cell membranes were incubated for 3 h at 4 °C with gentle rotation and then centrifuged at 20’000 *g* and 4 °C for 45 min. Cell membranes were also solubilized with the amphiphilic copolymer DIBMA and its derivative Carboxy-DIBMA at different polymer to membrane ratios of *R* = 0.25–1.0. For these polymers, cell membranes were incubated overnight at 4 °C with gentle rotation and then also centrifuged at 20 000 *g* and 4 °C for 45 min. Pellet and supernatant fractions were analyzed using sodium dodecyl sulfate-polyacrylamide gel electrophoresis (SDS-PAGE) and Western Blot analysis.

#### Protein precipitation for SDS-PAGE analysis

For SDS-PAGE analysis of proteins extracted by DIBMA, it is necessary to remove the polymer from the protein. Therefore, a 100 µL sample was mixed with 400 µL methanol, 100 µL chloroform, and 300 µL water. The mixture was then centrifuged at 14 000 *g* and 4 °C for 5 min. The upper phase was carefully removed without disturbing the white protein layer in the middle. Following this, 400 µL methanol was added, vortexed, and the solution was centrifuged at 5 000 *g* and 4 °C for 1 min, followed by centrifugation at 20 000 *g* and 4 °C for 4 min. The supernatant was discarded, and the protein pellet was dried using nitrogen. Subsequently, the protein pellet was resuspended in 1% SDS and used for electrophoresis experiments.

#### Sodium dodecyl sulfate-polyacrylamide gel electrophoresis (SDS-PAGE) analysis

SDS-PAGE samples were prepared by combining 20 µL of the sample with 4 µL of dithiothreitol (DTT, Stock 200 mM) and 8 µL 4 x SDS loading dye (8% SDS, 50% glycerol, 250 mM Tris-HCl, 0.02% bromphenol blue). The resulting mixture was vortexed, quickly centrifuged, and then heated to 90 °C for 10 min. For SDS-PAGE, Bolt Bis-Tris Plus Mini Protein Gels (4–12%, 1.0 mm, 15 well format) were used, and 10 µL of the sample was applied to the gel. The gel was run at a constant voltage of 200 mV and 150 mA for 45 min in precooled 1 x MES buffer. Western Blot analysis employed the PageRuler Prestained Protein Ladder (10 to 180 kDa). To assess the overall extraction efficiency of each compound, the gels were stained in Coomassie staining solution for 45 min and destained overnight in water. Images were captured using a ChemiDOC device with a White Sample Tray for Coomassie Blue, copper, silver, and zinc stains. SDS-PAGE analysis was performed by using ImageJ.

#### Western Blot analysis

After an SDS-PAGE run, the gels were subjected to dry blotting on a nitrocellulose membrane (Invitrogen, iBlot 2 Transfer Stacks, nitrocellulose) using the iBlot2 device with the P0 program. The membrane was immersed in 5% milk powder and resuspended in 5% BSA TBS-T buffer for 1 h at room temperature. Subsequently, the membrane underwent washing with TBS-T buffer and was then incubated overnight with a specific primary anti-TRPC3 antibody (TRPC3 (D4P5S) Rabbit mAb), diluted to 1:1 000 in 5% BSA TBS-T buffer. On the following day, the membrane was washed three times for 5 min each in TBS-T buffer and incubated for 1 h with the secondary HRP-conjugated goat anti-rabbit IgG antibody diluted to 1:10 000 in 5% BSA TBS-T buffer. After two 5-min washes with TBS-T buffer, the chemiluminescent signal for TRPC3 was detected using a ChemiDoc device with a UV Sample Tray using the Auto-optimale exposure option.

#### Protein purification

Supernatants containing TRPC3 from HEK293, *K. phaffii*, or Expi293F cells were transferred to a custom-made affinity chromatography column loaded with ANTI-FLAG^®^ M2-affinity gel. Proteins extracted with amphiphilic copolymers were incubated overnight under gentle rotation at 4 °C, whereas proteins extracted with DDDG or DDM were incubated for 3 h at 4 °C with gentle rotation. The column underwent a washing step with 20 column volumes (CV) of TBS buffer, and for detergents, TBS buffer was used along with 2 x CMC of each detergent. TRPC3 was eluted using 10 x CV of 3 x FLAG Peptide at a concentration of 100 µg/mL. The eluted fractions were combined and concentrated to approximately 150 µL using an Amicon Ultra-0.5 centrifuge filter unit with a molecular weight cutoff of 50 MWCO. The subsequent analysis involved SDS-PAGE, Western Blot analysis, negative stain electron microscopy (nsEM), electrophysiology experiments and targeted lipidomics.

#### Silver staining

For detecting contamination post-TRPC3 purification, the gel underwent a silver staining. First the gel was soaked in fixing solution (50% methanol, 13.5% formalin) for 10 min at room temperature. Subsequently, the gel was incubated overnight in water and, on the following day, incubated for 2 min in a sodium thiosulfate solution (0.02% sodium thiosulfate pentahydrate). Following a 2 × 20-second wash in water, the gel was then treated for 10 min in a 0.1% silver nitrate solution. Next, the gel was washed with a small amount of developing solution (3% sodium carbonate, 0.05% formalin, 0.000016% sodium thiosulfate pentahydrate) and incubated for 5–10 min until the desired band intensity was achieved. The reaction was stopped by incubating the gel in 2.3 M citric acid. Images were captured using a ChemiDoc device with a White Sample Tray.

#### Negative stain electron microscopy (nsEM)

To assess the purity and structure of purified TRPC3, negative stain transmission electron microscopy (nsTEM) experiments were conducted. A volume of 5–10 µL of the purified sample with a concentration of 0.1 mg/mL purified TRPC3 was deposited onto a TEM grid, allowed to incubate for 1 min, and subsequently removed from the grid. The grid was then stained with 1% uranyl acetate for 30 s before being imaged on a transmission electron microscope (Zeiss EM 900 with a Tröndle camera).

#### Lipidomics of purified TRPC3 samples with DDM and DDDG

Lipidomics experiments were conducted from 3 individual TRCP3 preparations of roughly 2 nmol purified protein. Lipids were extracted by Folch extraction. In brief, protein samples were incubated under shaking in 200 µL chloroform and 100 µL methanol for 10 min. 75 µL of water were added to induce phase separation, samples were vortexed and centrifuged for 5 min at 3 000 x g. The organic phase was transferred to HPLC vials and subjected to an untargeted lipidomics analysis. Lipids were separated on a Waters UPLC BEH C18 1 × 150 mm x 1.7 μm microbore column (Waters GmbH, Vienna, Austria) on a Vanquish H UPLC System (Thermo Fisher Scientific, Vienna, Austria) employing the following solvents and gradient: A: 60% acetonitrile, 15 mM ammonium formate. B: 90% isopropanol, 10% acetonitrile, 15 mM ammonium formate. Linear gradients between timepoints for both flow and % B: 0 min: 2% B, 100 µL/min; 15 min 100% B, 70 µL/min; 22 min 100% B, 70 µL/min; followed by equilibration to 2% B, 70 µL/min for 5 min. Mass spectrometric analysis was performed on a timsTOF HT (Bruker Daltonics GmbH, Bremen, Germany) equipped with a VIP-HESI source in both polarities, capillary voltage 4 500 V positive and 3 500 V negative mode. Probe Gas Temp was set to 150 °C, mobility range from 0.55 to 1.79 Vs/cm^2^ and mass range from 100 to 2 200 m/z with 2 MS/MS scans per full scan, total cycle time 0.32 s. Lipidomics data was analyzed employing Metaboscape 2022b (Bruker Daltonics GmbH, Bremen, Germany). Features were extracted using the T-Rex 4D algorithm and allowing ammonium adducts in positive and formic acid adducts in negative mode. Lipids were annotated using the rule based MCube Lipid Species annotation method with narrow windows of 2.0 ppm, 1% CCS deviation, greater 800 MS2 Score and milliSigma score below 50. Upper window boundaries were 5.0 ppm, 3% CCS deviation greater 400 MS2 Score and milliSigma score below 250.

#### Electrophysiology of purified TRPC3

To assess the activity of purified TRPC3, artificial membranes were generated in the Orbit Mini (Nanion, Germany) device using a MECA4 chip with a pore diameter of 100 µM. For this, 5 mg/mL DPhPC^60^ lipids were dissolved in Nonane and used to paint the membrane in 150 µL extracellular solution (150.3 mM potassium chloride, 3 mM magnesium chloride, 15 mM HEPES, pH = 7.4) at a temperature of 28 °C. The concentrated protein solution was centrifuged at 20,000 g and 4 °C for 30 min to separate aggregated TRPC3 from non-aggregated TRPC3. The concentration of the supernatant was then determined using Nanodrop measurements. Then, 4 µL of purified TRPC3 with a concentration of 0.15 mg/mL was transferred to the MECA4 chip and incubated for 25–30 min. Subsequently, 40 µL of intracellular buffer containing 137 mM sodium chloride, 5 mM potassium chloride, 2 mM calcium chloride, 2 mM magnesium chloride, and 10 mM HEPES at pH = 7.4 was added additionally to the 150µL extracellular solution. Voltages of 80 mV was applied at a frequency of 5 kHz, with a signal-to-noise ratio of SR2. Additionally, 0.4 µL of GSK from a stock concentration of 10 mM was added, and recordings were obtained.

Negative controls were conducted at + 80 mV, examining various conditions: the membrane response solely to voltages, to DMSO, to 20 µM GSK, to 0.4 mM DDM, to 0.2 mM DDDG, and also the response of TRPC3 to DMSO. Furthermore, we inhibited the channel post-activation by applying 40 µM of Pyr10^65^.

#### Electrophysiology of TRPC3 in adherent HEK293 cells

TRPC3 activity in adherent HEK293 cells was investigated via whole-cell and single-channel patch clamp experiments conducted 24 h post-transfection, as described in a prior publication^64^. An inverted microscope (Zeiss Axiovert 200 M, Germany) equipped with a 40 × 0.75 objective was used, with a CoolLED pE-300 ultra (CoolLED, UK) serving as the excitation source. Transfected cells were identified by illuminating at a wavelength of 490 nm for the YFP-Tag and detecting emission at 515 nm.

#### Whole-cell configuration

For whole-cell analysis of TRPC3 activity, patch-clamp recordings were conducted in whole-cell configuration. Patch pipettes were fabricated from thin-walled borosilicate glass (Clark Electromedical Instruments, UK) using a pipette puller (Sutter Instruments, CA, USA). Signals were low-pass filtered at 2 kHz and digitized at 8 kHz. Linear voltage-ramp protocols ranging from − 130 to + 90 mV (holding potential 0 mV) were applied using Clampex 11.0 (Axon Instruments) software. Current densities at − 90 and + 70 mV were plotted against time and normalized by capacitance. For pharmacological measurements, cells were maintained at room temperature and perfused with GSK (10 µM in DMSO) in extracellular solution (ECS) containing 140 mM NaCl, 10 mM HEPES, 10 mM Glucose, 2 mM MgCl_2_, and 2mM CaCl_2_, pH adjusted to 7.4 with NaOH. The pipette solution (Intracellular solution, ICS) comprised 150 mM cesium methanesulfonate, 20 mM CsCl, 15 mM HEPES, 5 mM MgCl_2_, and 3 mM EGTA, adjusted to pH 7.4 with CsOH. Thin-wall capillary pipettes made from borosilicate glass with a filament (Harvard Apparatus, USA) were pulled to a resistance of 2–5 MΩ using a pipette puller (Sutter Instruments, CA, USA).

#### Single channel configuration

For single-channel analysis of TRPC3 activity, thin-wall capillary pipettes made from borosilicate glass with a filament (Harvard Apparatus, USA) were pulled to a resistance of 15–20 MΩ using a pipette puller (Sutter Instruments, CA, USA). Currents were recorded at room temperature using the Axopatch 200 B amplifier (Molecular Devices, USA). Single-channel activity was recorded in the cell-attached configuration. Thick-wall capillary pipettes made from borosilicate glass with a filament (Harvard Apparatus, USA) were pulled to a resistance of 10–15 MΩ using a pipette puller (Sutter Instruments, CA, USA).

The Extracellular Solution (ECS) contained: 145 mM potassium gluconate, 5.3 mM KCl, 3 mM MgCl_2_, and 15 mM HEPES. The Intracellular Solution (ICS) contained 137 mM NaCl, 5 mM KCl, 2 mM CaCl_2_, 2 mM MgCl_2_, and 10 mM HEPES. The pH value of all solutions was adjusted to pH 7.4. Cells were stimulated with 10 µM GSK. For inhibiting TRPC3 activity, 20 µM of Pyr10 was used, mixed with 10 µM of GSK. Experiments were conducted at room temperature.

Single-channel currents were digitized at a sampling rate of 50 kHz and filtered with the Axopatch-200B internal 4-pole low-pass Bessel filter (–3 dB cut-off at 2 kHz). The holding potential ranged from + 80 mV and was controlled by the holding command function of the amplifier. Data acquisition, analysis, and further filtering with a low-pass Gaussian filter (−3 dB cut-off at 1.5 kHz) were performed using Clampfit 11 software (Axon Instruments, Foster City, CA), followed by histogram analysis in the Python-based tool SCANA.

For the analysis of amplitude distribution histograms in SCANA, a tool was implemented in Python V3.9.1 utilizing the scientific computing packages NumPy V1.19.5 and SciPy V1.6.0, as well as the plotting package Matplotlib. For the analysis, the following model:

$$\:f\left(x\right)=\:\sum\:_{i=1}^{n}{\alpha\:}_{i}\text{*}\text{e}\text{x}\text{p}(-({\left(x-{\beta\:}_{i}\right)*{\gamma\:}_{i})}^{2})$$

Was used with parameters α_i_, β_i_, and γ_i_ for i = 1, …,n and an arbitrary but fixed n. To fit the model to the histogram a curve fitting method based on a non-linear least squares problem was applied to optimize the set of model parameters. From the fitted parameters α_i_, β_i_, and γ_i_, mean values µ_i_ = β_i_, standard deviations σ_i_ = (2γi^2^)^−0.5^, and scaling factors ρ_i_ = $\:\sqrt{\pi\:*{\gamma\:}_{i}^{-1}*{\alpha\:}_{i}}$can be derived and the model can be written as sum of scaled normal distributions:

$$\:f\left(x\right)=\:\sum\:_{i=1}^{n}{\rho\:}_{i}*\frac{1}{{\sigma\:}_{i}\sqrt{2\pi\:}}\text{e}\text{x}\text{p}(-\frac{1}{2}({\frac{x-{\mu\:}_{i}}{{\sigma\:}_{i}})}^{2})$$

which simplifies the interpretation of the parameters.

#### Statistics analysis

Data analysis and graphical representation were conducted using Clampfit 11 (Axon Instruments, USA), SCANA, and Origin2022b. Results are presented as mean values ± SEM. Statistical significance for normally distributed data was evaluated using both unpaired and paired Student’s *t*-tests. For non-normally distributed data, the paired Wilcoxon rank test was used. In general, differences were considered significant at * *p* < 0.05, ** *p* < 0.01, and *** *p* < 0.001.

## Supplementary Information

Below is the link to the electronic supplementary material.

Supplementary Material 1

## Data Availability

The data will be available upon request. Contact Oleksandra Tiapko, Email: oleksandra.tiapko@medunigraz.at.
