# Supplementary material for: Utilizing native nanodiscs to isolate active TRPC3 channels and expand structural analysis capabilities
Source: Sci Rep. 2025 Aug 5;15:28562. doi: 10.1038/s41598-025-13218-6 (PMC12325699; doi:10.1038/s41598-025-13218-6)
Supplement: Supplementary file 1 — Supplementary Material 1 [file 41598_2025_13218_MOESM1_ESM.docx]

**Supplemental Information**


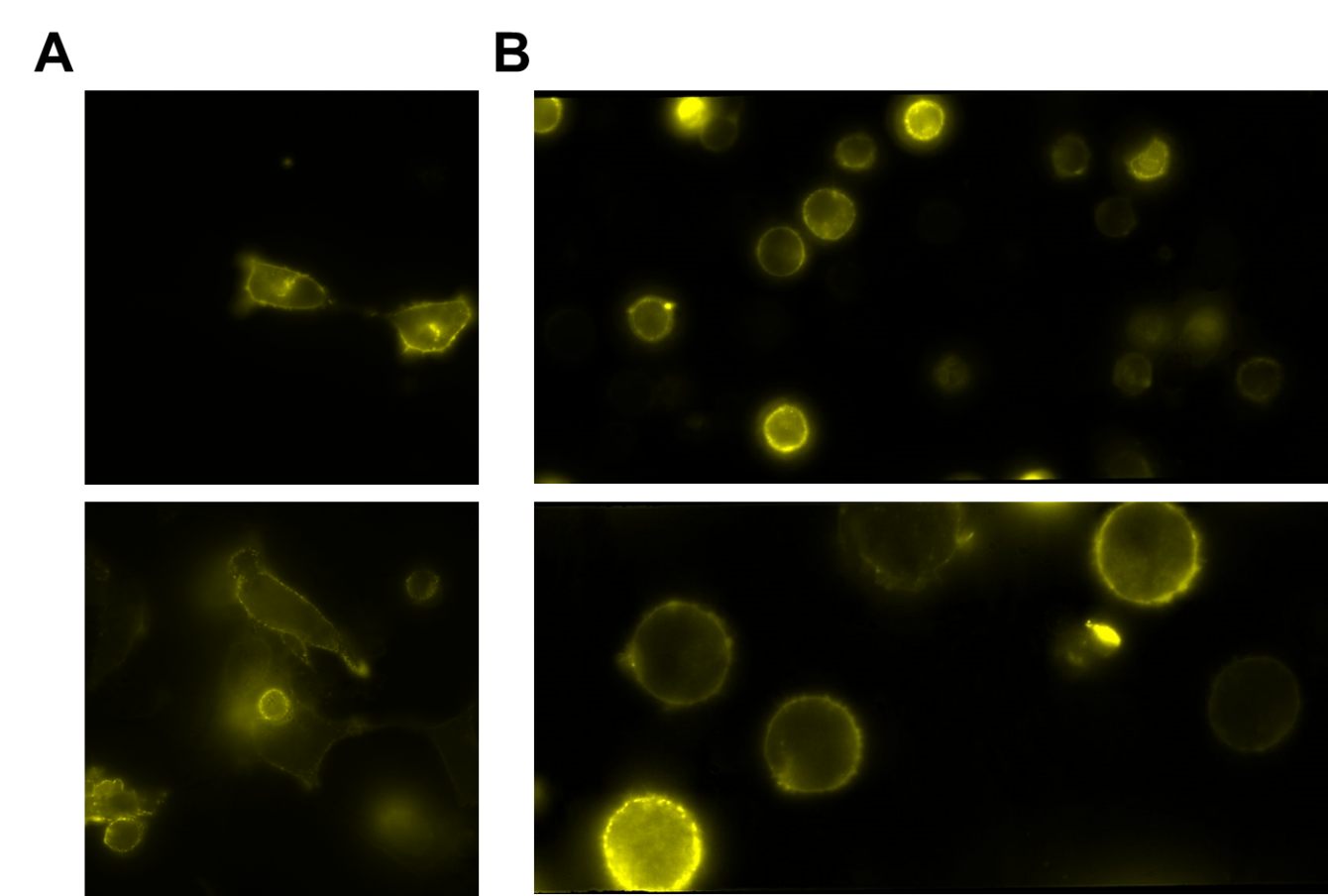


**Figure S1: Overexpression of TRPC3-YFP-3xFLAG in A) adherent HEK293 cells and B) Expi293F cells. A)** TRPC3 is overexpressed in adherent HEK293 cells and predominantly localized to the plasma membrane. Both images were captured at a 100x magnification. **B)** Similarly, TRPC3 is overexpressed in Expi293F cells, with primary localization in the plasma membrane. The upper image was captured at 40x magnification, while the lower image was acquired at 100x magnification. Both expression systems demonstrate TRPC3 overexpression at the plasma membrane, as indicated by the YFP signal.


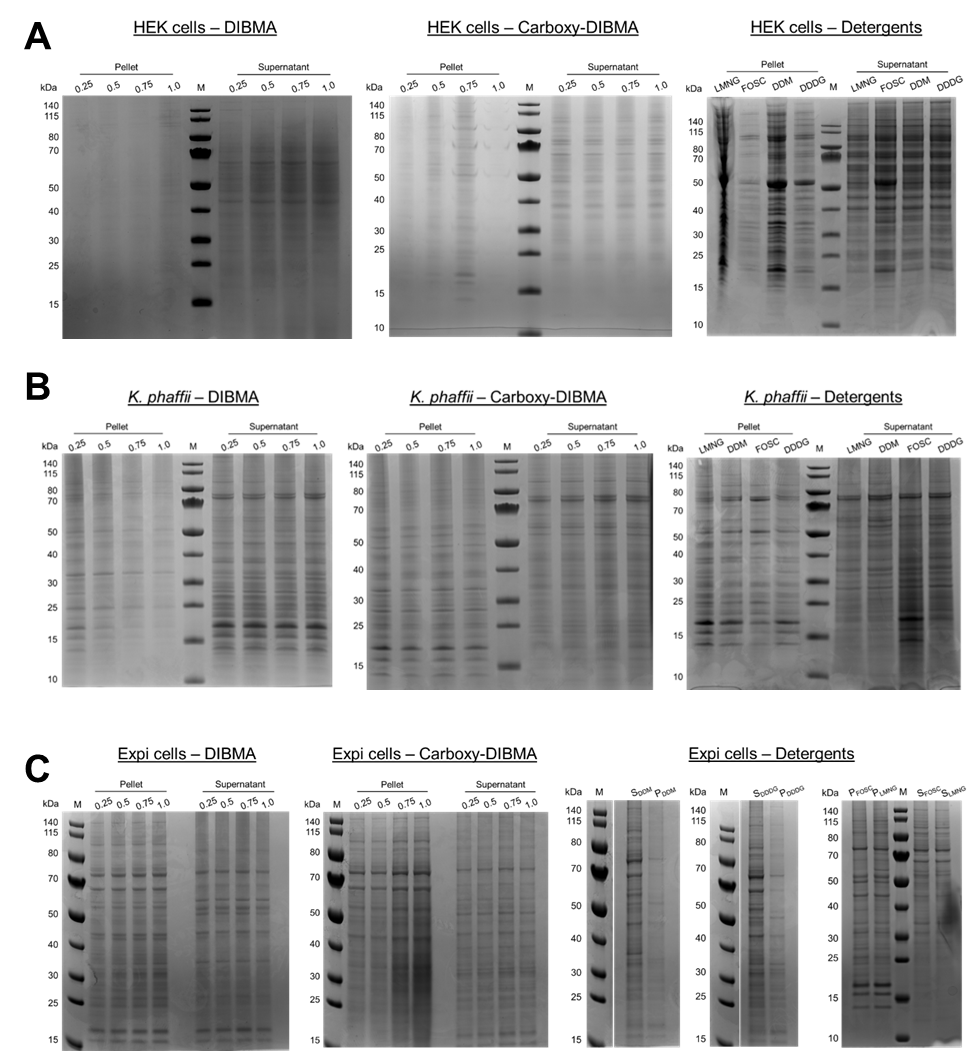


**Figure S2: SDS-PAGE analysis of the extracted membrane proteins from each expression system.** SDS-PAGE analysis was conducted on all supernatant (S) and pellet (P) fractions derived from 25 mg/mL solubilized membrane samples obtained from **A)** HEK293 cells, **B)** *K. phaffii*, and **C)** Expi293F cells using DIBMA, Carboxy-DIBMA, DDM, DDDG, FOS-Choline (FOSC), and LMNG. Please note that the right side of Panel C was cropped. The uncropped SDS-PAGE can be found in Supplementary Information, Figure S7.


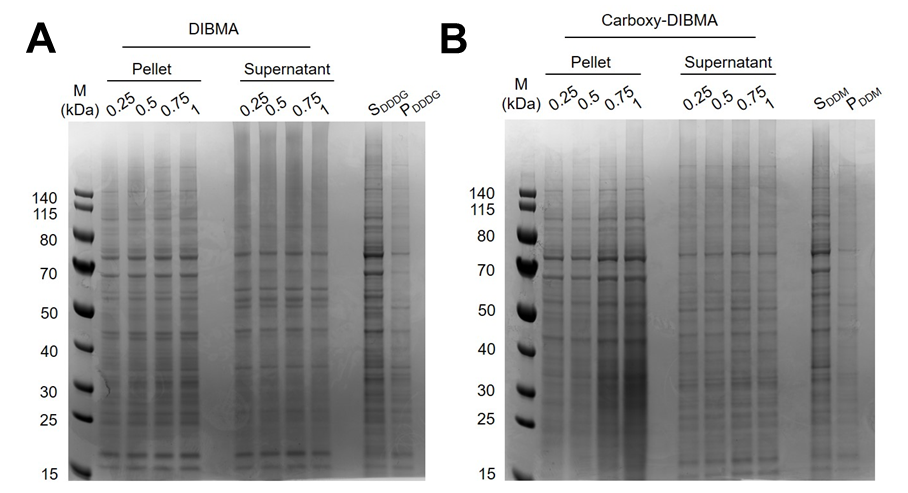


**Figure S3:** **SDS-PAGE analysis of the extracted membrane proteins from Expi293F cells.** SDS-PAGE analysis was conducted on all supernatant (S) and pellet (P) fractions derived from 25 mg/mL solubilized membrane samples using DIBMA (**A**), DDDG (**A**), Carboxy-DIBMA (**B**), and DDM (**B**).


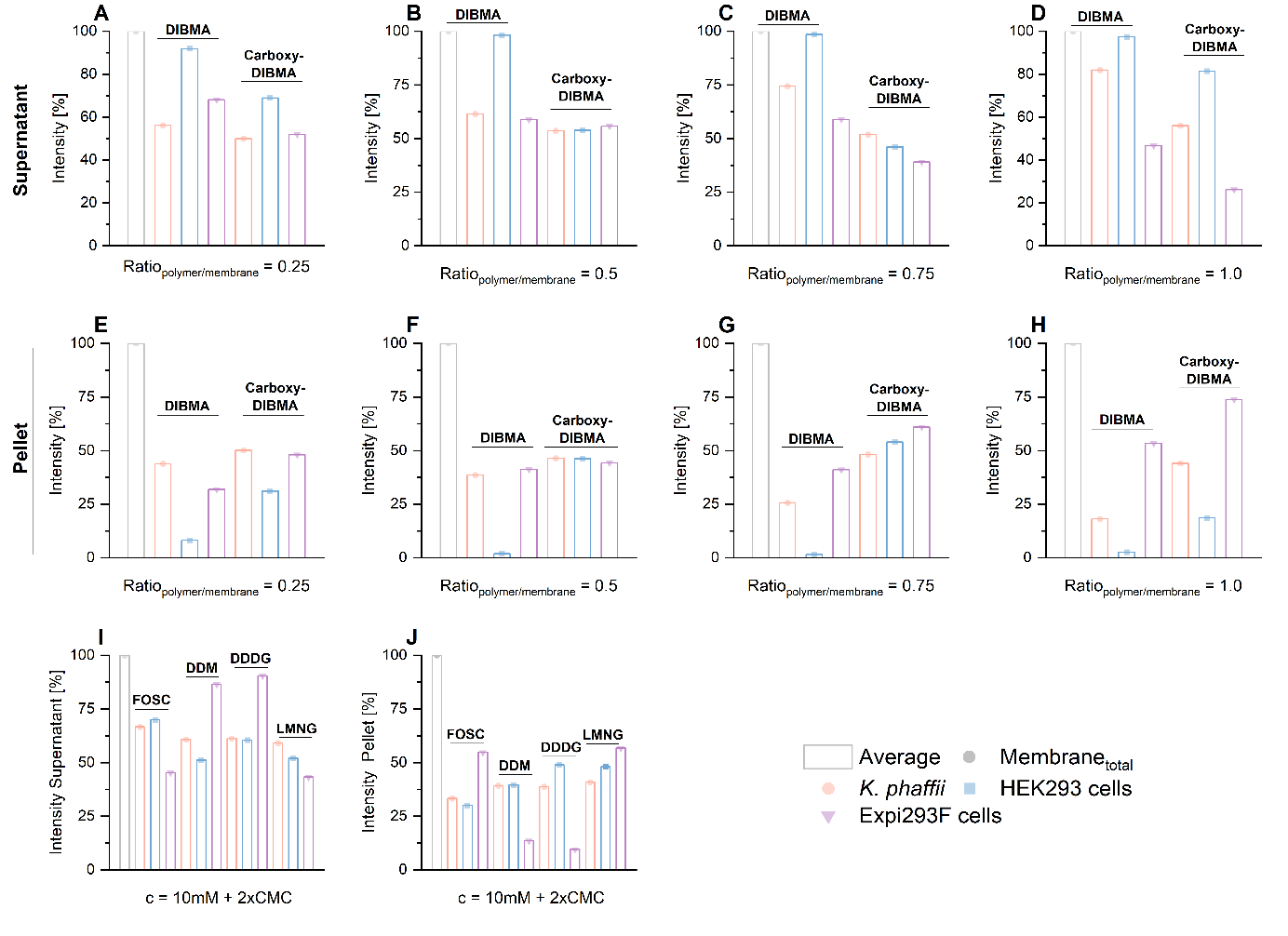


**Figure S4: Extraction of the membrane proteins from each expression system.** ImageJ analysis was conducted on all **A–D**, **I**) supernatant and **E–H, J**) pellet fractions derived from 25 mg/mL solubilized membrane samples obtained from adherent HEK293 (blue), *K. phaffii* (orange), and Expi293F cells (purple) using DIBMA, Carboxy-DIBMA, DDM, DDDG, FOS-Choline, and LMNG. For DIBMA and Carboxy-DIBMA, different polymer/membrane ratios of *R* = 0.25–1.0 were used, while for detergents a concentration of 2 x CMC + 10 mM was used.


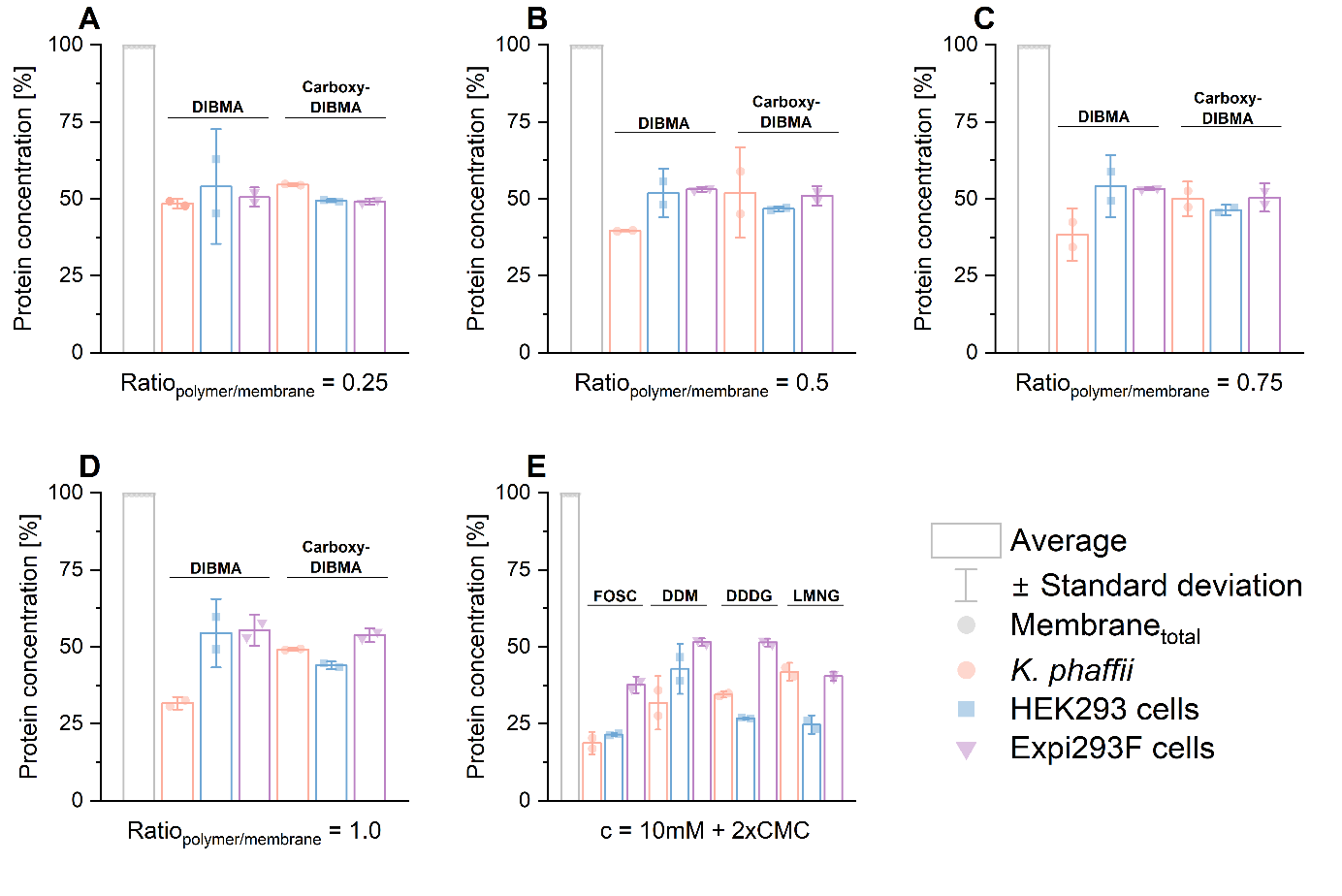


**Figure S5: BCA-Assay analysis of the remaining membrane proteins in the pellet fractions.** BCA assay analysis was conducted on all pellet fractions derived from 25 mg/mL solubilized membrane samples obtained from adherent HEK293 (blue), *P. pastoris* (orange), and Expi293F cells (purple) using DIBMA, Carboxy-DIBMA, DDM, DDDG, FOS-Choline, and LMNG. The membrane protein concentration was normalized to the total membrane protein concentration and expressed as a percentage (%). **A–D)** Protein concentrations of remaining membrane proteins with DIBMA and Carboxy-DIBMA at different polymer/membrane ratios: *R* = 0.25 (**A**), *R* = 0.5 (**B**), *R* = 0.75 (**C**), and *R* = 1.0 (**D**). **E)** Protein concentrations of remaining membrane proteins with FOS-Choline (FOSC), DDM, DDDG, and LMNG with 2 x CMC + 10 mM of each detergent.


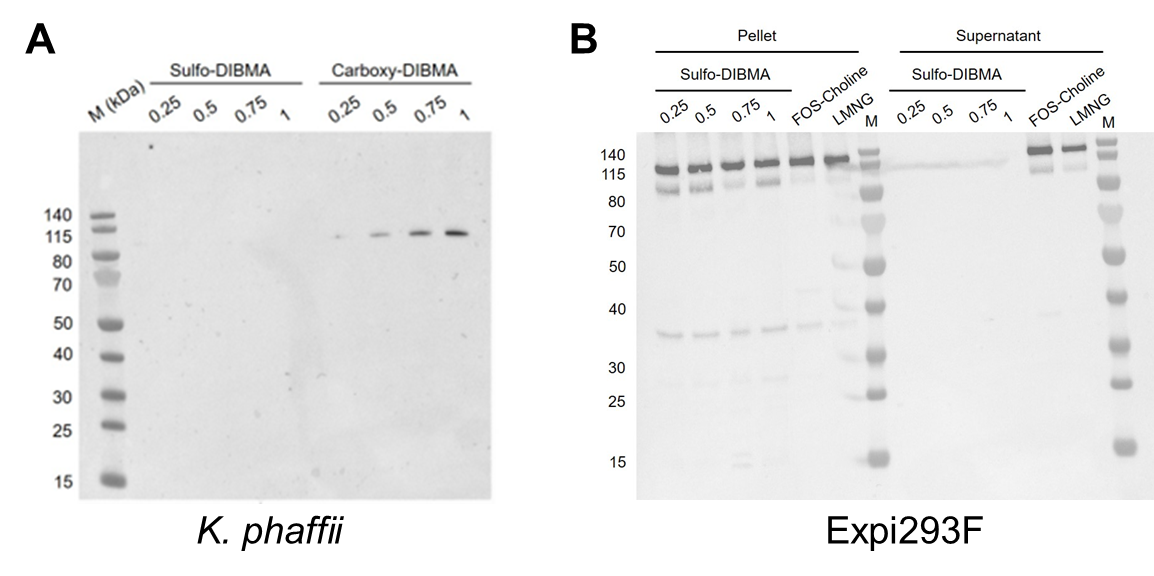


**Figure S6: Western Blot analysis of supernatant fractions containing all membrane proteins extracted with Sulfo-DIBMA, Carboxy-DIBMA, LMNG or FOS-Choline (FOSC) from K. phaffii (A), and Expi293F suspension cells (B).** Western Blot analysis was conducted on the supernatant fractions extracted from adherent K. phaffii (**A**), and Expi293F (**B**) cell membranes using Sulfo-DIBMA and Carboxy-DIBMA at various polymer-to-membrane ratios ranging from R = 0.25 to 1.0, as well as FOS-Choline (FOSC), and LMNG. A specific TRPC3 antibody was used to target TRPC3.


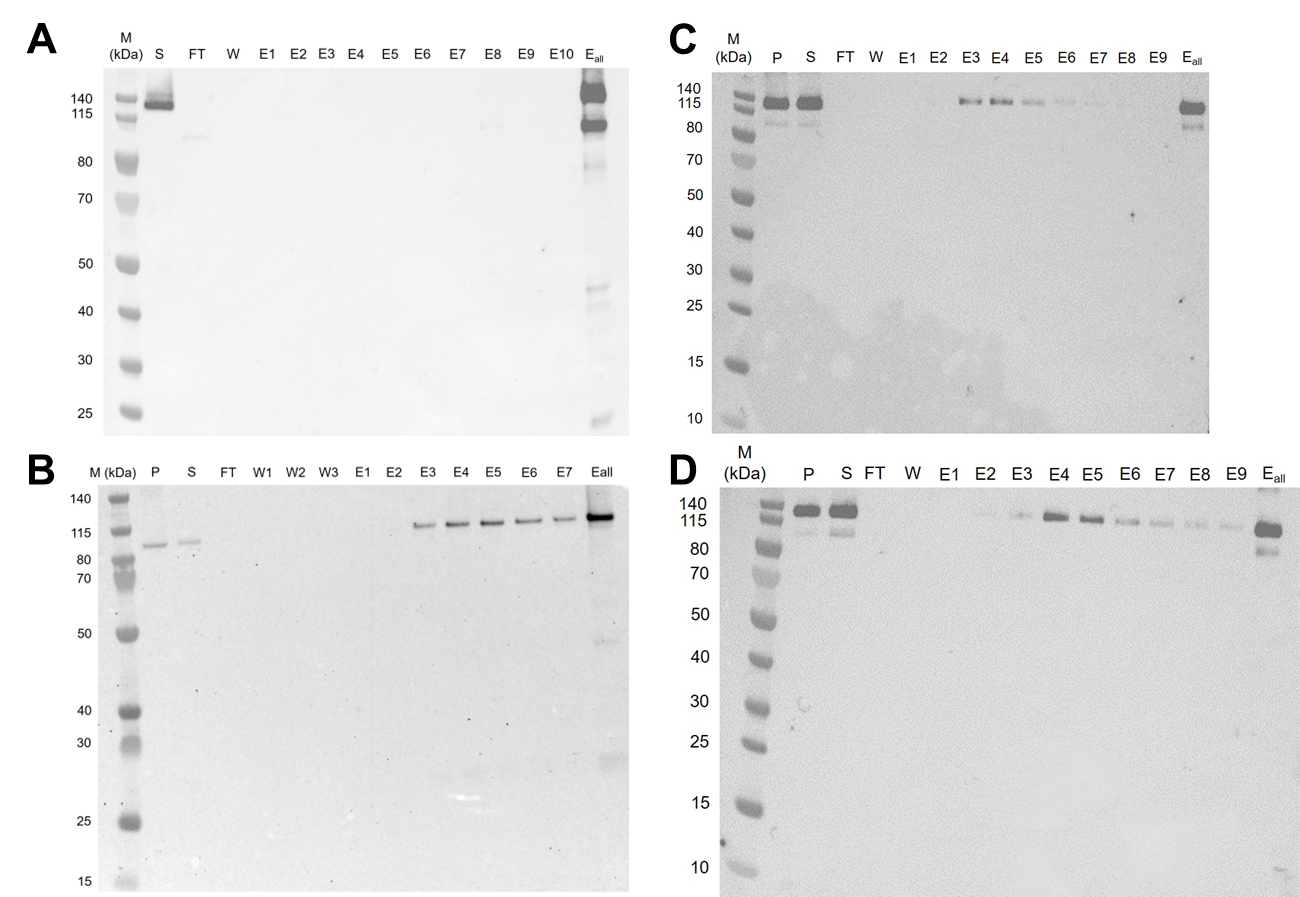


**Figure S7:** **Western blot analysis of purified TRPC3.** TRPC3 was purified from adherent HEK293 cell membranes using DIBMA (A), *K. phaffi* cell membranes using DDM (*n* = 3, **B**), and Expi293F cell membranes using DDDG (*n* = 6, **C**) or DDM (*n* = 6, **D**).


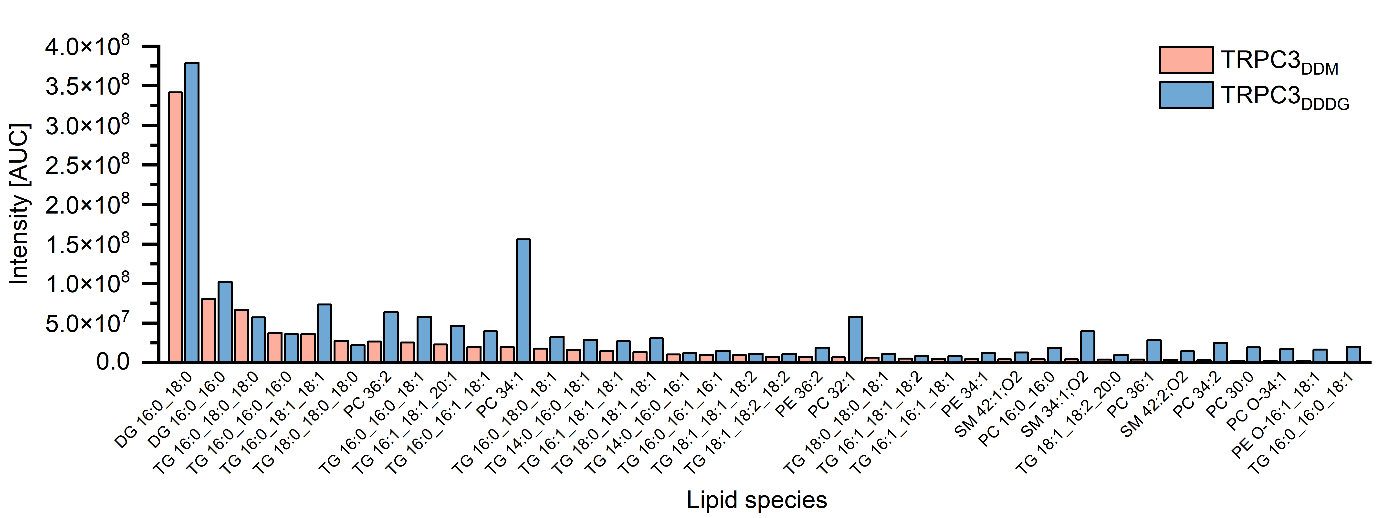


**Figure S8: Lipidomics analysis of TRPC3 samples purified with DDM (orange) and DDDG (blue).** In both purified TRPC3 samples different types of lipids were found especially diacylglycerol (DG), triglycerides (TG), phosphocholine (PC) and phosphatidylethanolamine (PE).


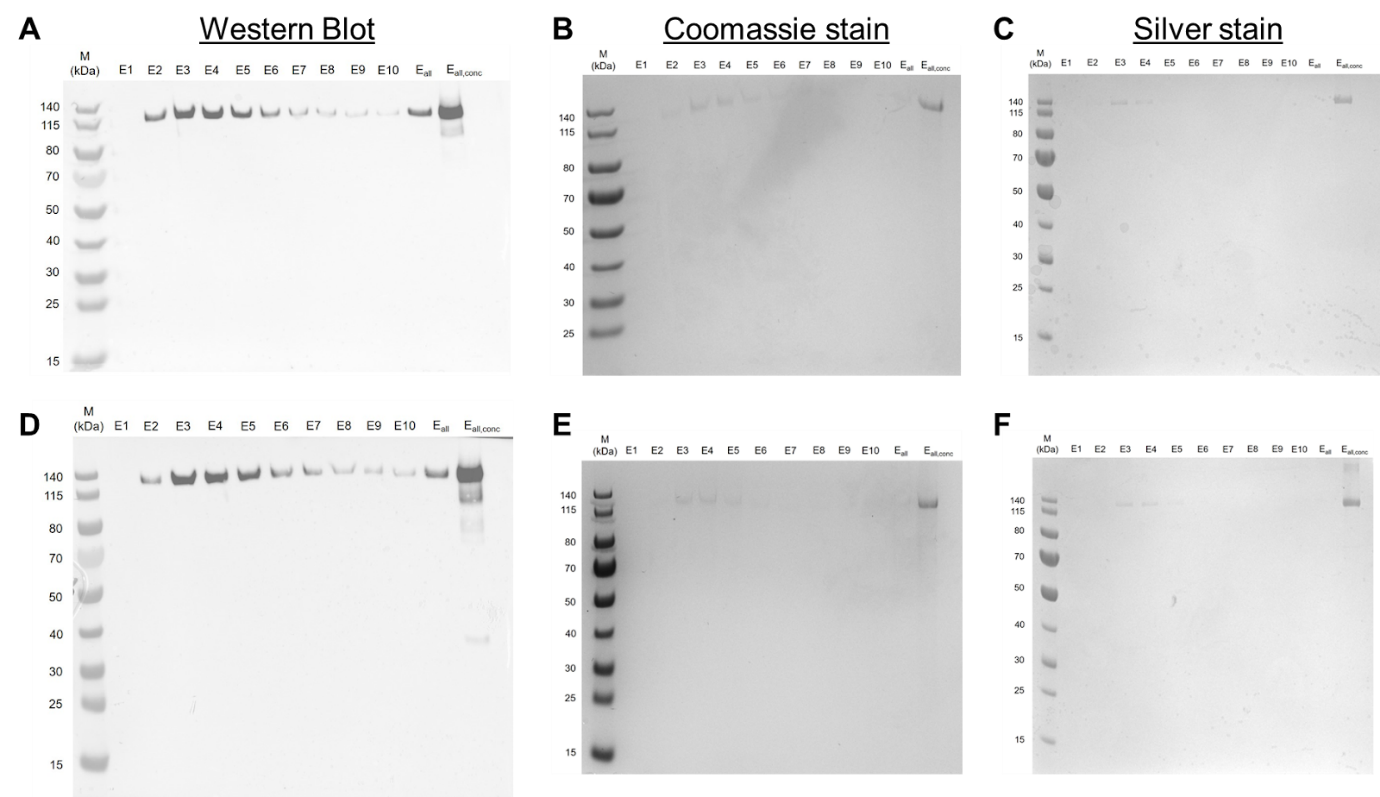


**Figure S9: Successful purification of TRPC3 with DDDG (A–C) or DDM (D–F) without co-purification of other proteins. A, D)** Western Blot analysis of purified TRPC3, **B, E)** Coomassie-stained SDS-PAGE analysis of purified TRPC3 and **C, F)** Silver stain SDS-PAGE analysis of purified TRPC3 from 175 mg of Expi293F cell membranes.


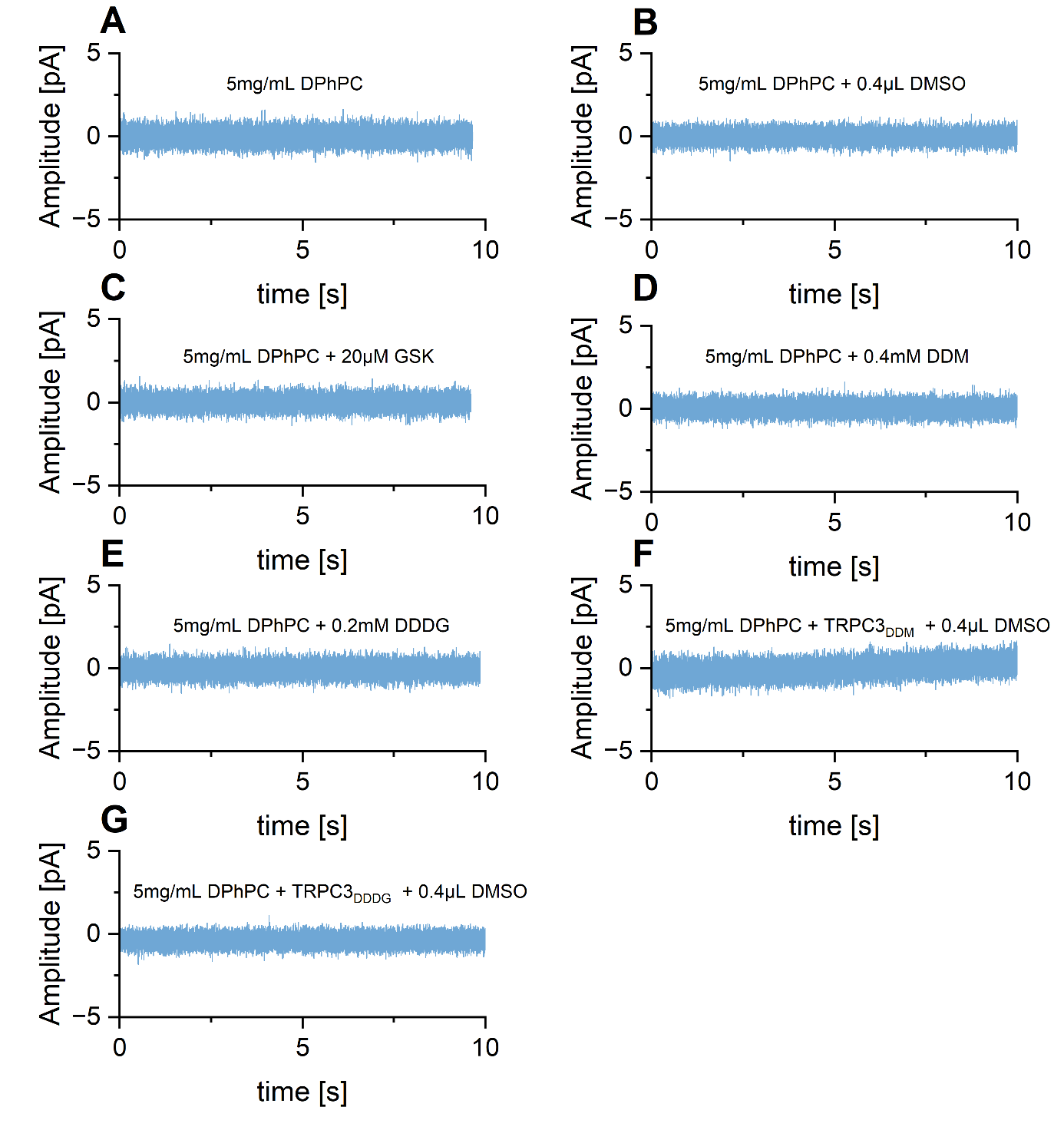


**Figure S10: Control experiments showed no disturbance of the lipid bilayer by solvent or detergents.** The DPhPC membrane was challenged **A)** without or with **B)** DMSO (0.4 µL), **C)** GSK (20 µM), **D)** DDM (0.4 mM), and **E)** DDDG (0.2 mM) while clamped at +80 mV. Purified TRPC3 with **F)** DDM and **G)** DDD was reconstituted into the membrane, and DMSO (0.4 µL) was applied while the bilayer was clamped at +80 mV.


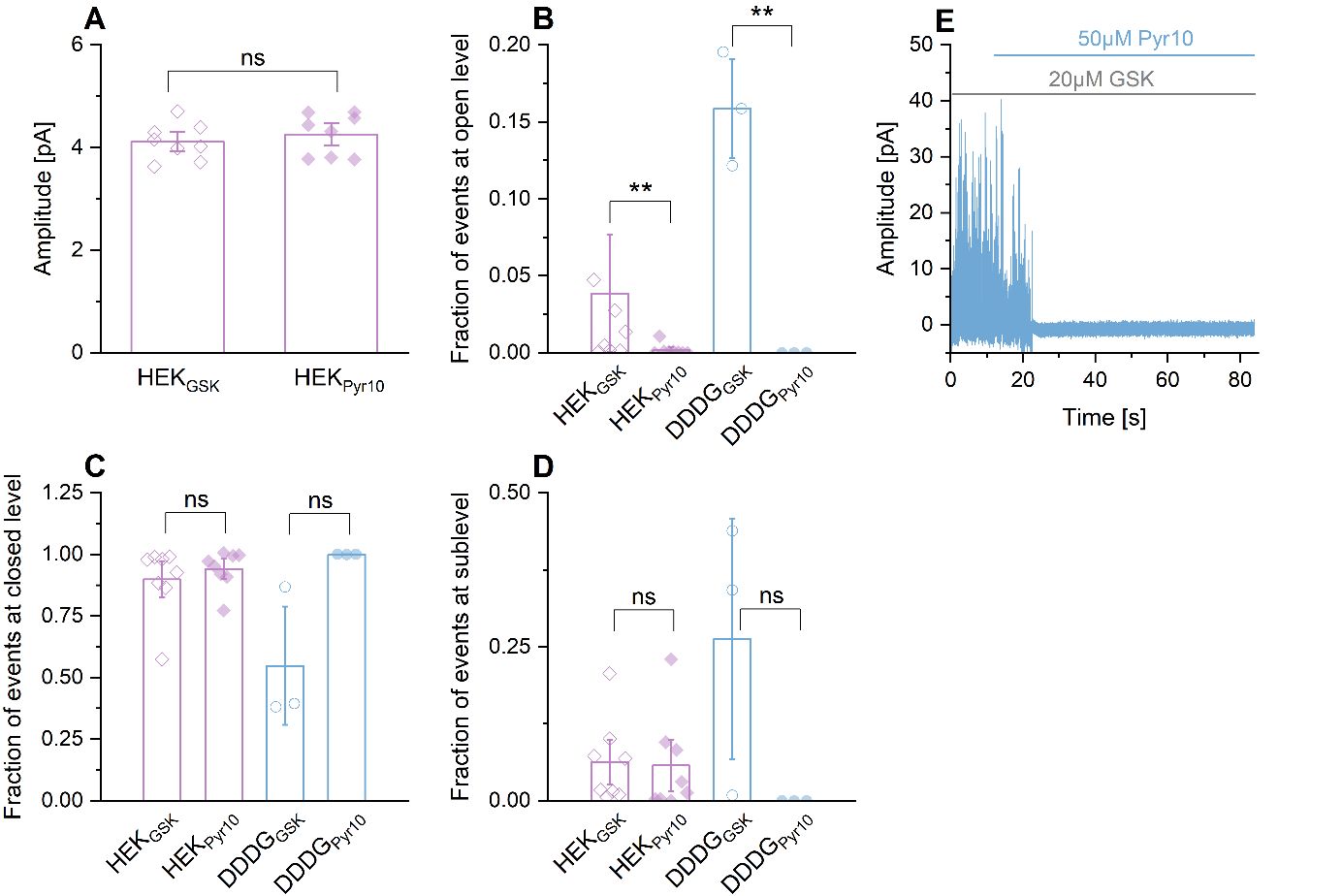


**Figure S11: Pyr10 effectively inhibits TRPC3 activity in both HEK293 cells and when the channel is reconstituted in the lipid bilayer. A)** Unitary currents corresponding to the open level were recorded from adherent HEK293 cells expressing TRPC3 (purple) and reconstituted TRPC3 purified with DDDG (blue) in response to applications of GSK (10 µM for adherent HEK293 cells or 20 µM for reconstituted TRPC3) and Pyr10 inhibitor (20 µM in adherent HEK293 cells or 40 µM in reconstituted TRPC3) at a membrane potential of +80 mV. Data are presented as means ± SEM. Statistical significance was evaluated using paired two-tailed multiple t-tests for normally distributed data and the Wilcoxon test for non-normally distributed data. ns = not significant. The fraction of events derived from Gaussian fitting for the **B)** open, **C)** closed, and **D)** sub-levels of single-channel behavior recorded from adherent HEK293 cells expressing TRPC3 (purple), and reconstituted TRPC3 purified with DDDG (blue), in response to applications of GSK (10 µM for adherent HEK293 cells or 20 µM for reconstituted TRPC3) or Pyr10 (20 µM for adherent HEK293 cells or 40 µM for reconstituted TRPC3) at a holding potential of +80 mV. Data are presented as means ± SEM. Statistical significance was assessed using a two-tailed multiple t-test and Wilcoxon test. ns = not significant. **E)** Representative trace depicting TRPC3 inactivation by Pyr10 after channel activation with GSK.


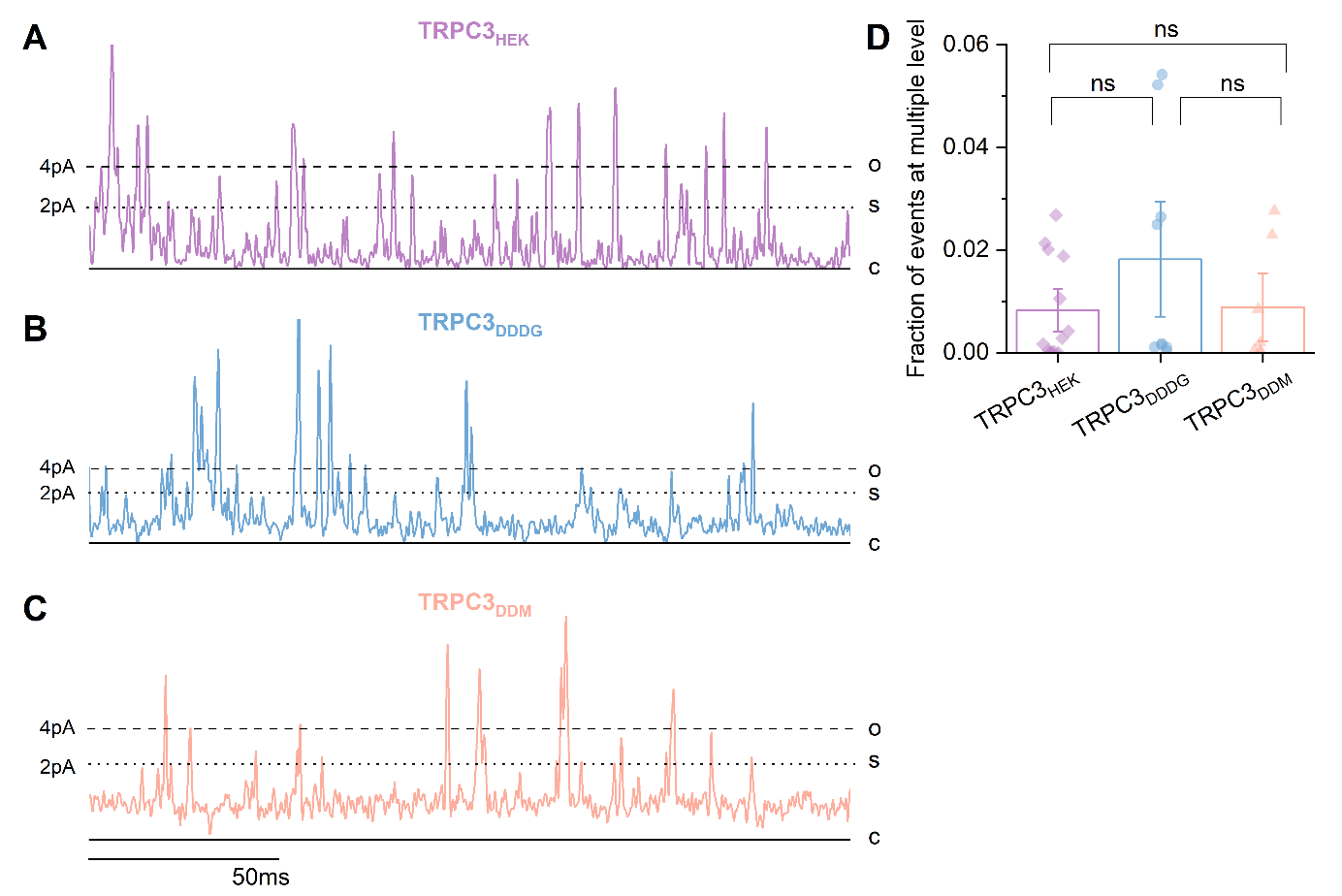


**Figure S12: Representative single-channel currents showing 200 ms of GSK-induced TRPC3 activity.** **A-C)** TRPC3 was either expressed in A) HEK293 cells (purple) or purified from Expi293F cells using B) DDDG (blue) or C) DDM (orange) and reconstituted into DPhPC bilayers. GSK was applied at 10 μM for adherent HEK293 cells and 20 µM for reconstituted TRPC3. Currents were recorded at +80 mV. Channel states are indicated as closed (c), sublevels (s), or open (o). D) Fraction of events at multiple levels derived from Gaussian fitting of single-channel recordings from HEK293 cells expressing TRPC3 (purple), reconstituted TRPC3 purified with DDDG (blue) or with DDM (orange). GSK was applied at 10 μM for HEK293 cells and 20 µM for reconstituted TRPC3 at a membrane potential of +80 mV. Data are presented as means ± SEM. Statistical significance was assessed using a two-tailed multiple t-test or the Wilcoxon test, with differences considered significant at * p < 0.05, ** p < 0.01, and *** p < 0.001. Non-significant differences are indicated as "ns".
